# Targeting Cellular Senescence in Aging and Age-Related Diseases: Challenges, Considerations, and the Emerging Role of Senolytic and Senomorphic Therapies

**DOI:** 10.14336/AD.2024.0206

**Published:** 2024-02-27

**Authors:** Liyao Zheng, Shipei He, Hong Wang, Jinling Li, Yuanyuan Liu, Sijia Liu

**Affiliations:** ^1^Collaborative Innovation Centre of Regenerative Medicine and Medical BioResource Development and Application Co-constructed by the Province and Ministry, Guangxi Key Laboratory of Regenerative Medicine & Key Laboratory of Longevity and Aging-related Diseases of Chinese Ministry of Education, Guangxi Medical University, Nanning, Guangxi, China.; ^2^Guangxi Colleges and Universities Key Laboratory of Biological Molecular Medicine Research & Guangxi Key Laboratory of Brain Science, Department of Biochemistry and Molecular Biology, School of Basic Medical Sciences, Guangxi Medical University, Nanning, Guangxi, China

**Keywords:** aging, age-related diseases, senolytics, senomorphics, senescent cells

## Abstract

Cellular senescence is characterized by the permanent arrest of cell proliferation and is a response to endogenous and exogenous stress. The continuous accumulation of senescent cells (SnCs) in the body leads to the development of aging and age-related diseases (such as neurodegenerative diseases, cancer, metabolic diseases, cardiovascular diseases, and osteoarthritis). In the face of the growing challenge of aging and age-related diseases, several compounds have received widespread attention for their potential to target SnCs. As a result, senolytics (compounds that selectively eliminate SnCs) and senomorphics (compounds that alter intercellular communication and modulate the behavior of SnCs) have become hot research topics in the field of anti-aging. In addition, strategies such as combination therapies and immune-based approaches have also made significant progress in the field of anti-aging therapy. In this article, we discuss the latest research on anti-aging targeting SnCs and gain a deeper understanding of the mechanism of action and impact of different anti-aging strategies on aging and age-related diseases, with the aim of providing more effective references and therapeutic ideas for clinical anti-aging treatment in the face of the ever-grave challenges of aging and age-related diseases.

## 1.Introduction

Cellular senescence was first proposed by Hayflick in the 1960s [1]. Cellular senescence and aging of organisms are closely related processes, and aging is a natural and gradual process that can be predicted by organisms. It is caused by cell death or senescence due to DNA damage, peroxidation, protein misfolding, and other pro-aging mechanisms. Aging is a stress response, the early stages of which may be due to one or more of the factors such as telomere erosion, DNA damage, oxidative stress, oncogene activation, mitochondrial dysfunction, radiation, or drugs [2]. It is a process of growth arrest in which the cell is essentially stable and irreversible in response to various stressors [3-5]. During this phase, nuclear chromatin remodeling and loss of nuclear lamina protein B1, mitochondrial oxidative metabolism, and the SnC anti-apoptosis pathways (SCAPs) are activated in SnCs. With the increase in lysosomal activity, the activity of senescence-associated secretory phenotype β-galactosidase (SA-β-gal) increases and induces the secretion and release of senescence-associated secretory phenotype (SASP) [6]. Among them, SASP is an important feature of SnCs, including the release of many cytokines, chemokines, growth factors, and proteases into the extracellular environment [6]. The continuous accumulation of SnCs induces an inflammatory state in the body through the secretion of SASP [7].

SnCs accumulate in most organ tissues with age [8, 9]. Increasing evidence suggests that excessive accumulation of SnCs in the body triggers tissue remodeling, organismal aging, etc., which ultimately leads to the development of age-related diseases, and that lifespan can be prolonged and age-associated diseases can be prevented or delayed by removing SnCs [10, 11]. In mammals, age-related degeneration contributes to the development of many diseases, such as sarcopenia, atherosclerosis, heart failure, osteoporosis, macular degeneration, renal failure, and neurodegenerative diseases, as well as many more age-related pathological changes [12-18]. Aging is the largest risk factor for impaired cardiovascular health, and cardiovascular disease is the cause of death in 40% of people over the age of 65 [19]. Recently, Xia et al. combined the concepts of aptamer-mediated active cell recognition and multiple marker co-activation of prodrugs to construct drugs that can target a broad spectrum of senescent endothelial cells [20]. The designed drug efficiently removed senescent endothelial cells and foamy macrophages in an atherosclerosis model, thereby reducing plaque area and effectively inhibiting plaque progression in an atherosclerosis model [20]. Alzheimer's disease (AD) is an age-related neurodegenerative brain disorder, with the greatest risk factor being aging. The majority of AD patients are diagnosed by people over 65 years of age. Previous studies have found that clearing SnCs is associated with reduced AD pathology (i.e., tau protein and Aβ deposition), reduced SASP and inflammation, reduced white matter hypersignaling, restoration of cerebral blood flow, and improvement in cognitive behavior [21]. Joseph et al. found that the proportion of neurons expressing aging markers in the brains of AD patients increased significantly and showed metabolic dysfunction and pro-inflammatory features [22]. The secretory phenotype related to neuronal aging also induces astrocyte proliferation [22]. Therefore, drugs targeting senescence-like neurons may be a strategy to prevent or treat AD. The hyperglycemia and metabolic changes of diabetes can promote cell senescence, which in turn leads to multiple diabetic complications [23]. Drugs that remove SnCs improve blood glucose levels and reduce diabetic complications [24]. In addition, diabetes leads to cardiac stem cell senescence, and Marino et al. found that cardiac function could be improved in diabetic mice by eliminating SnCs in a mouse model of type 2 diabetes, which is accompanied by cardiomyopathic remodeling and cardiac dysfunction [25]. Therapeutic strategies targeting SnCs may also contribute to prolonging the healthy lifespan, with studies showing that bone marrow-derived mesenchymal stromal cells (MSCs) can improve the function of senescent stem cells and senescent fibroblasts and prolong the lifespan of mice [26].

As promising therapeutic strategies for the treatment of age-related diseases, the elimination of SnCs as well as the reduction of SnCs-associated secretory phenotypes remain hot research topics at the forefront of current medicines [27]. Senomorphics and senolytics are emerging areas of anti-aging research. Senomorphics are compounds that alter the behavior of SnCs, while senolytics are compounds that selectively eliminate SnCs. Because of their potential to be selected for SnCs, these compounds have received a lot of attention from researchers and are being used in the field of anti-aging research. With the discovery of small molecules and anti-aging drugs that selectively remove SnCs, many promising strategies have been provided for the prevention and treatment of a variety of human and age-related diseases. However, the use of one drug alone is limited for the treatment of age-related diseases, and multi-drug combination therapies are gradually emerging. Combination therapy can reduce the side effects of single drugs and the development of resistance in the body, so it is reasonable and effective to apply combination therapy to SnCs. With the increase in age, the body will inevitably show a decline in immune system function and the accumulation of SnCs, and a large number of studies have shown that both can significantly accelerate aging [28-32]. Therefore, immune cell therapy, small molecules, and anti-aging drugs that selectively clear SnCs have become the focus of many researchers in the field of anti-aging. To sum up, the acceleration of cellular aging is often accompanied by age-related diseases, such as cardiovascular diseases, neurodegenerative diseases, and metabolic diseases. Targeted clearance or inhibition of SnCs can slow the progression and deterioration of these diseases, improve tissue function, reduce inflammatory responses, reduce cancer risk, improve immune function, and extend a healthy life span. However, the safety issues, potential side effects, and ethical issues brought about by targeting SnCs cannot be ignored, which are also challenges that need to be overcome in the future anti-aging road.

In this review, we describe the role of SnCs in the aging process and explain the key role SnCs play in a variety of diseases, such as cancer, neurodegenerative diseases, diabetes, joint diseases, cardiovascular diseases, liver diseases, etc. At the same time, we highlight the therapeutic mechanisms and recent advances of senolytics and senomorphics, two important compounds in the field of targeting SnCs in aging and age-related diseases. In addition, we describe the therapeutic role of immunotherapy targeting SnCs for aging and age-related diseases in animal and clinical trials. On the basis of these clarifications, we further reveal the possible challenges and directions for improvement of therapeutic approaches targeting SnCs in future clinical practice. Overall, this review provides a comprehensive and in-depth overview of the importance of targeting SnCs in addressing aging and age-related diseases, provides a clear picture of the development and potential clinical implications of the field, and seeks to provide a strong guide for future research and clinical practice. In conclusion, therapeutic strategies targeting SnCs represent an innovation in the field of geriatrics, offering the possibility of finding new ways of treating age-related diseases and being of great significance for the development of anti-aging pathways.

## 2.Senescence and Aging

### 2.1 The role of cellular senescence in aging

Cellular senescence is a complicated cellular state triggered by stressful damage and certain physiological processes (e.g., DNA damage, oncogene activation, oxidative stress, or exogenous toxicant exposure) and is characterized by prolonged and usually irreversible cell cycle arrest due to SASP, macromolecular damage, and metabolic alterations [33, 34]. The process of cell proliferation arrest due to cellular senescence occurs gradually as telomere shortening and DNA damage accumulate [35]. The division of proliferatively active cells is regulated by signaling pathways that operate at different stages of the cell cycle. Cell cycle arrest, which can occur at G1, G2, or G0 (outside the cell cycle), is indispensable for resolving the complete phenotype of senescence-GS by preventing cells from entering the division phase with catastrophic consequences in the presence of DNA damage or insufficient bioenergetic resources [36, 37].

As one of the key driving factors among the 12 hallmarks of aging, cellular senescence is a driving force for organ and organism aging [38, 39]. Biomarkers of cellular senescence can be used not only to monitor cellular-level changes during aging but also to indicate the biological age of an entire organ or organism [39]. The most compelling evidence for the role of cellular senescence in aging is that sustained genetic or pharmacological elimination of SnCs extends the lifespan of naturally senescent mice [40]. Both excessive generation and insufficient elimination of SnCs may lead to pathological senescence [41].

### 2.2 The molecular mechanisms of cellular senescence

DNA damage, telomere dysfunction, oncogene activation, and organelle stress are some of the common factors that induce cellular senescence [42, 43]. The first molecular feature associated with senescence is telomere shortening. The formation of G-quar double-stranded bodies at telomeres from G-rich strands may lead to replication stagnation and collapse [44]. This is a consequence of end-DNA replication (i.e., the DNA replication mechanism is unable to complete the synthesis of the very top of the linear chromosome, and telomeres shorten with cell division) [45]. Telomere shortening also occurs through oxidative damage and other end-processing events in dividing and non-dividing cells [46]. Telomere dysfunction caused by telomere shortening, telomere structural collapse, or displacement of the telomere protective protein complex elicits a DNA damage response that leads to loss of cell proliferation, senescence, or apoptosis [46]. Nuclear DNA damage has also been reported to be a common underlying cause of senescence, manifested primarily by DNA double-strand breaks that activate the DNA damage response pathway [47]. The presence of DNA and other types of macromolecular damage ultimately leads to proliferative arrest through the activation of the p53/p21^CIP1^ and p16INK4a/RB tumor suppressor pathways [48]. In the case of irreparable damage, cells may be forced to die or definitively exit the cell cycle, thereby ceasing replication or segregating chromosomes with unrepaired damage [37].

Inflammatory factors in SASP are key links between SnCs and inflammation, mainly including pro-inflammatory interleukin 6 (IL-6), IL-8, and monocyte chemoattractant protein 1, which are regulated in an IL-1-dependent manner, and enzymes involved in extracellular matrix remodeling [43]. The content of SASP varies according to cell type, external stimuli, and environmental background [49, 50]. SASP represents the characteristics of SnCs and has a significant impact on aging and age-related diseases. Factors secreted by SnCs can enhance the senescence phenotype in an autocrine manner, but they can also affect neighboring cells in a paracrine manner [51]. SnCs trigger their fibrotic effects mainly through the secretion of SASP and pro-fibrotic factors, which have been observed in the context of skin wounds or senescent fibroblast accumulation after myocardial infarction, which functionally limits tissue fibrosis [52].

In addition, mitochondrial dysfunction also plays an important role in cell senescence and growth arrest, promotes the development of SASP, and inhibits cell death [53]. At the same time, some researchers have found that mitochondrial Ca^2+^ overload can lead to a decrease in mitochondrial membrane potential and an increase in reactive oxygen species (ROS) production, thus leading to aging and age-related diseases [54, 55]. In addition, numerous studies have shown that cellular senescence is characterized by significant changes in mitochondrial dynamics and histology. It is generally accepted that damaged mitochondria can fuse with healthy mitochondria, dilute and rearrange the matrix, or utilize fission to separate and subsequently degrade damaged mitochondria [53]. In addition to nuclear DNA, mitochondrial DNA (mtDNA) is also a target of exogenous or endogenous stressors that result in mutations and deletions that may lead to aging and cancer [53].

### 2.3 Senescent cells cause age-related changes

It has been reported that as long as the stress injury is below the level of cellular lethality, cells survive and enter the senescent state rather than dying through programs such as apoptosis or necrosis [56]. Cellular senescence is generally considered an anticancer mechanism because it limits the cell's potential for division [57] and can prevent cells from unnecessary damage and genetic destabilization [58]. Senescence occurs in all cell types at any stage of life, from embryogenesis to adulthood, but mainly in fibroblasts, endothelial cells, and immune cells [59, 60]. Cell senescence is a type of programmed death in which the number of SnCs increases with age and occurs in many types of cells in most tissues (including lymphatic tissue) [28, 61, 62].

SnCs are considered "hallmarks of cancer" and may be associated with tumorigenesis, such as oncogene-induced or treatment-induced senescence [63]. Although the early senescence response promotes T cell recruitment and tumor cell clearance, it is possible that T cell suppressive properties may be acquired at a later stage after senescence induction [63]. Several recent studies have explored in detail how SnCs promote anti-tumor immune responses, but others have reported that SnCs enhance tumor resistance to immunotherapy through powerful immunosuppressive mechanisms. Therefore, the aging of tumor cells does not simply mean the surrender of tumor cells but is more like a "double-edged sword" [64, 65]. Cellular senescence prevents initial tumorigenesis in a cell-autonomous manner, but the accumulation of SnCs in tissues stimulates the development of chronic inflammation, transforming the tumor into an "unhealable wound" [66].

Normally, SnCs are cleared by the immune system soon after their appearance, but with chronic stress or immune dysfunction, SnCs can persist and develop more pro-inflammatory, pro-apoptotic SASP [67, 68]. Animal experimental studies have shown that impaired immune cytotoxicity can lead to the accumulation of SnCs [69]. Therefore, immune function may be one of the factors influencing the rate of SnC accumulation. Between SnCs and host immunity, intracellular events associated with aging initiation affect organismal function in a non-cellular autonomic manner through the release of senescent secretory bodies or extracellular vesicles [70]. The release of SASP mediators lays the foundation for the termination of the senescence-driven tissue response. This is achieved through the chemical attraction of immune cells responsible for eliminating SnCs. Due to a considerable degree of environmental dependence, both innate and adaptive immune cells can keep SnCs under surveillance [41]. Pharmacological or genetic interventions that trigger senescence in the most malignant cells limit tumor growth in a natural killer (NK)- and T-cell-dependent manner [71]. Significant accumulation of senescence macrophages has also been confirmed in human lung cancer samples, and it has been found that specific subpopulations of macrophages in tissues can promote tumorigenesis by altering their local microenvironment [72]. Recognition and elimination of SnCs by specialized immune cells is a critical step in maintaining tissue homeostasis and preventing adverse inflammation [62]. SnCs also take up neutrophils, and tissue infiltration of neutrophils is one of the hallmarks of immune senescence [62]. Researchers have found that the senescence of immune cells not only leads to impaired immune function but also contributes to damage to other non-immune organs and shortens the lifespan of mice. Immune cell senescence may promote systemic aging, and the key to preventing aging is to maintain the number and activity of immune cells in the body [62]. Transplantation of senescent immune cells can also promote senescence and tissue damage [62]. Conversely, transplantation of "young" immune cells can reverse aging to a certain extent [62]. T cells regulate inflammation and drive and play a key role in age-related diseases. Pro-inflammatory T-cell subsets (e.g., Th1 and Th17 cells) typically promote organismal aging, whereas Treg cells are more likely to suppress inflammatory responses and rejuvenate the body [73].

## 3.Senescence and Age-Related Diseases

As a result of social and dietary influences, human lifespan is gradually increasing, but with it, the prevalence of age-related diseases is snowballing and is now one of the leading causes of morbidity and mortality worldwide [74]. Immune senescence leads to impaired immune function, thereby reducing the clearance of SnCs [62, 69]. One study found that the elimination of SnCs in elderly mice reduces the level of systemic inflammatory mediators, indicating that SnCs are a key source of inflammation [11]. Conversely, transplanting SnCs into young mice increased systemic inflammation and led to physical dysfunction [40]. At the same time, SASP secreted by SnCs can adversely affect neighboring cells, the surrounding extracellular matrix, and other structural components, leading to chronic inflammation and inducing the senescence of healthy cells and fragile tissues [75, 76]. Removing SnCs or inhibiting SASP can alleviate or delay a variety of chronic age-related diseases, which also demonstrates the therapeutic potential of targeting SnCs [11, 40, 62]. Several months after cells enter senescence, transposons (elements such as LINE1) can be produced within SnCs along with reverse transcriptase. These elements lead to further activation of intracellular DNA rearrangement and DNA damage signals, thereby inducing the intracellular cGAS-STING damage response pathway and strengthening and further aggravating the production of pro-inflammatory and pro-apoptotic SASP [67]. In Ercc1-/Δ mice, elimination of NF-κB-dependent SASP delays the onset of progeria-like symptoms and prolongs lifespan [77]. Notably, transplanting small amounts of SnCs into young, healthy animals reproduced age-related impairments in physical function [78]. This supports the threshold hypothesis, which states that once the SnCs burden increases in tissues beyond sustainability, it activates age-related pathological changes and ultimately leads to disease [10]. Genetic clearance of p16INK4a-high SnCs in the INK-ATTAC mouse model has demonstrated the benefits of SnC clearance in preventing or mitigating diseases, including osteoporosis, frailty, atherosclerosis sclerosis, hepatic steatosis, osteoarthritis, idiopathic pulmonary fibrosis, obesity-induced anxiety, Tau-mediated neurodegenerative diseases, and type 2 diabetes/metabolic dysfunction [10, 21, 79-81]. It can be seen that the continuous accumulation of SnCs in organs and tissues will lead to the occurrence of many age-related diseases, such as cancer, neurodegenerative diseases, diabetes, cardiovascular diseases, osteoarthritis, and liver diseases.

### 3.1 Cancer

Research suggests that SnCs may play a role in the progression of cancer. The accumulation of SnCs is associated with tumor formation and growth. Previous studies have confirmed that chronic inflammation is one of the most important drivers of cancer occurrence [76, 82]. Zhang et al. found that cellular senescence changes the pericellular microenvironment, thereby driving the release of substances such as inflammatory factors and extracellular matrix enzymes to promote tumor growth and inhibit immune responses to tumors [83-85]. A number of SASP factors have been shown to be associated with pro-tumorigenic processes, including chronic inflammation, mitogenic signaling, stemness, angiogenesis, migration, and invasion [86, 87]. Among them, IL-6 and IL-8 are known drivers of cancer proliferation [88, 89]. SnCs promote the transformation of non-malignant cells into malignant ones [90], and D-galactose can induce accelerated aging models [91]. Researchers have been exploring ways to target and eliminate SnCs in cancer therapy, aiming to selectively kill SnCs while preserving normal, healthy cells [92, 93]. This therapeutic strategy of selectively removing SnCs as a therapeutic strategy is expected to improve the overall health of tissues and reduce the risk of cancer development and progression.

### 3.2 Neurodegenerative disease

There is a correlation between cellular senescence and neurodegenerative diseases. Neurodegenerative diseases are a group of disorders characterized by neurological dysfunction, such as AD and Parkinson's disease (PD). These diseases are usually associated with cellular senescence and damage. In AD, cellular senescence may be associated with abnormal deposition of β-amyloid and damage to neuronal synapses. PD is associated with the degeneration of dopamine neurons and damage to substantia nigra cells, which may be related to oxidative stress and mitochondrial dysfunction. The presence of SnCs has been linked to the onset and progression of neurodegenerative diseases. It has been found that SnCs accumulate in large amounts in the brain tissue of AD patients. These cells may secrete inflammatory mediators and toxic proteins, leading to impaired neuronal function and increased neuroinflammatory responses. Studies have shown that the phenotypes associated with aging endothelial cells contribute to the development of some neurological diseases, such as AD [94]. In the aging brain, astrocytes lose their ability to perform normal functions and release a toxic factor that kills neurons and oligodendrocytes [95]. Both processes directly affect the integrity of the transgenic tissue and lead to structural decline in vulnerable brain regions during normal aging. Recent evidence on cellular senescence reveals that senescence may be one of the underlying mechanisms of complex cellular responses during the progression of AD [96]. Herdy et al. found that the proportion of neurons expressing senescence markers was significantly higher in AD brains [22]. In addition, SnCs have been detected in the brains of AD patients and AD model mice that overexpress Aβ or tau proteins [21, 97]. In these AD model mice, the removal of SnCs pharmacologically and genetically reduced brain Aβ deposition and tau lesions and improved memory [21]. Therefore, elucidating the mechanism of brain cell aging in AD and the mechanism of neurodegeneration caused by SnCs will be the key to finding prevention and treatment strategies for age-related diseases such as AD [98]. Activation of the BMP4-SMAD1/5/9-RUNX2 pathway will inhibit the neurogenesis and oligodendrogenesis of iPSCs in AD patients with age-related diseases [99]. Upregulation of p38MAPK, a well-known SASP regulator, and an increase in SASP factors such as the pro-inflammatory cytokines IL-6, IL-1β, TGF-β, and TNF-α, as well as the extracellular proteases MMP-1, MMP-3, and MMP-10, were observed in AD brains [100-104]. Brain samples were obtained from older adults and AD patients after death, and it was found that AD patients had a significantly higher proportion of senescent astrocytes in the frontal cortex compared to normal age-matched individuals. Also, induction of primary cultured astrocytes with Aβ1-42 in vitro was found to cause senescence in a concentration-dependent manner [105]. When rat astrocytes were cultured, they showed signs of aging, including strong SA-β-gal staining, elevated ROS production, and bioenergy deficits, which impaired their ability to maintain neurons and adversely affected the aging brain [106, 107]. It has been found that after Aβ injury, there is an increase in SA-β-gal-positive cells, an increase in senescence-associated proteins P16 and P21, and a decrease in energy-related molecules (ATP, NAD^+^, NAD^+^/NADH) and mitochondrial function (mitochondrial ROS, MMP) in BV2 cells [108]. Selective elimination of senescent astrocytes inhibited the development of paraquat-induced neurodegenerative phenotypes associated with PD, suggesting that astrocyte senescence may be a key mechanism of neurodegeneration in PD [109]. Considering that senescence is one of the major factors contributing to PD, animal studies have been performed and found that the upregulation of signature proteins in astrocytes is age-dependent [110]. It was also found that during normal aging, astrocytes in the substantia nigra and hippocampus (a region of the brain susceptible to degenerative disease) appeared to have greater changes in RNA expression than during normal aging [110]. The cGAS-STING-YY1 axis can promote the aging of astrocytes by up-regulating the expression of LCN2 and further promoting the progression of PD [111]. These findings all suggest the role of astrocytes in age-related chronic diseases such as PD. In summary, SnCs play an important role in the occurrence and development of neurodegenerative diseases such as AD and PD.

### 3.3 Diabetes

Diabetes-induced cell senescence can lead to various diabetic complications [23]. In the pathogenesis of type 2 diabetes, insulin resistance causes compensatory proliferation of β cells. Due to the limited replicative potential of β cells, this compensatory proliferation may accelerate cellular senescence and lead to diabetes [112]. It has been shown that in a mouse model of type 1 diabetes, the absence of senescent β cells enhances insulin secretion and preserves insulin secretory capacity, thus establishing a new link between cellular senescence and severe insulin deficiency [113]. Aguayo-Mazzucato et al. found that senescent β cells were increased and showed dysfunction of insulin secretion in aged mice compared to normal controls, and that sensitivity to high-glucose stimuli was significantly reduced [114]. This study also showed that insulin resistance induced an increase in the expression of pancreatic β cell senescence markers such as p16INK4a, IGF-1R, bone-forming proteins, and transmembrane inhibitor of activin (Bambi) and accelerated β cell senescence [114]. Minamino et al. found that excessive caloric intake could increase oxidative stress in mouse adipose tissue and lead to cell aging, including increased SA-β-gal activity, p53 expression, and increased production of pro-inflammatory cytokines [115]. Inhibition of p53 activity in adipose tissue could significantly improve these aging-like changes and alleviate insulin resistance [115]. Inhibition of SnCs or their products in adipose tissue has been shown to improve metabolism in aged mice [79].

### 3.4 Cardiovascular diseases

The presence of SnCs is closely related to the development of cardiovascular diseases. Studies have shown that SnCs accumulate in atherosclerotic plaques and participate in plaque formation and rupture by secreting substances such as inflammatory factors and extracellular matrix enzymes [116]. The presence of SnCs may lead to the inflammatory response of the artery wall and the decline of plaque stability, increasing the risk of cardiovascular events [116, 117]. Atherosclerotic plaques display cellular senescence characterized by decreased cell proliferation, irreversible growth arrest and apoptosis, elevated DNA damage, epigenetic modifications, and telomere shortening and dysfunction [118]. Cellular senescence is not only associated with atherosclerosis, but there is increasing evidence that cell senescence promotes atherosclerosis [118]. Asymptomatic progression of atherosclerotic plaque can lead to major cardiovascular disease, including acute myocardial infarction or ischemic stroke in some cases [119]. Different cellular senescence can produce different pathophysiological effects, among which the senescence of endothelial cells, vascular smooth muscle cell, and immune cells is strongly correlated with atherosclerosis [117, 120, 121]. Cellular senescence accelerates atherosclerosis by promoting endothelial activation through epigenetic alterations, and Honda et al. showed that endothelial cell senescence plays a crucial pathogenic role in the progression of atherosclerosis in vivo [122]. Active pro-atherosclerotic mechanisms are present in SnCs of senescent vessels, and senescent endothelial cells have been shown to have lower nitric oxide (NO) production and increased expression of the adhesion molecules vascular cell adhesion molecular-1 and intercellular adhesion molecule 1, which bind monocytes to contribute to endothelial infiltration [123].

### 3.5 Osteoarthritis

The ability of chondrocytes to proliferate and synthesize declines with age, and this complex disease involves a variety of different effects ranging from inflammatory mediators to epigenetic alterations. Cellular senescence may play an important role in the pathology of osteoarthritis [124]. Increased numbers of SnCs in articular cartilage have been found to correlate with increased pain and inflammatory responses, and these cells may contribute to the development of osteoarthritis by disrupting joint structure and function through the secretion of inflammatory factors and enzymes that degrade joint tissues [125]. Farr et al.’s analysis showed that aging bone marrow cells and bone cells up-regulated the production of a variety of key SASP factors with age [126]. In addition, age-related mitochondrial dysfunction and associated oxidative stress may induce joint tissue cell senescence [127]. Transplantation of a small number of SnCs around the knee joint in young mice leads to age-related osteoarthritis-like disease [78]. Aging is key to the pathogenesis of osteoarthritis and may be interdependent with other risk factors, including obesity, joint damage, and genetic susceptibility [126, 128]. Aging is correlated with the number of senescent articular chondrocytes and the progression of osteoarthritis [129], suggesting that targeting SnCs could be a key breakthrough in preventing or reversing osteoarthritis processes.

**Figure 1.**
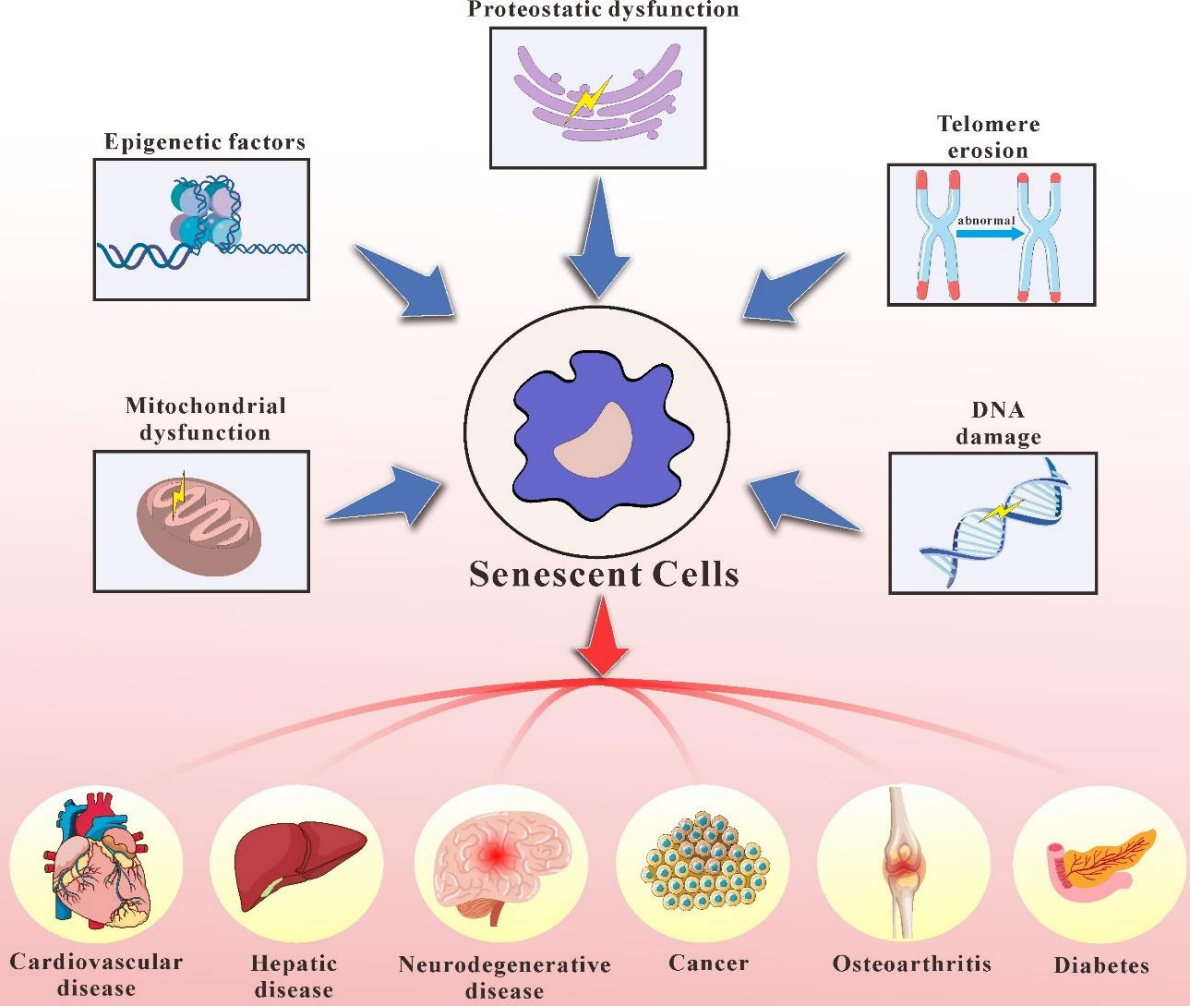
**Major triggers of cellular senescence and age-related diseases triggered by the accumulation of excessive SnCs**. Mitochondrial disfunction, epigenetic factors, proteostatic dysfunction, telomere erosion, and DNA damage are all factors that contribute to cellular senescence, while the excessive accumulation of SnCs leads to a wide variety of diseases in the body (e.g., cardiovascular disease, hepatic disease, neurodegenerative disease, cancer, osteoarthritis, diabetes, etc.).

### 3.6 Liver disease

The relationship between cellular senescence and liver disease can be explained by a variety of mechanisms. One common mechanism is that cellular senescence leads to increased inflammation in liver tissue. Aging of the liver may lead to decreased liver function, increasing the risk of chronic liver disease and liver cancer [130]. Through histological analysis of the liver tissues of young and old cynophagous monkeys, researchers found that the livers of old monkeys showed aging phenotypes such as increased SnCs, elevated inflammatory factors, increased fibrosis, lipid deposition, and decreased heterochromatin [131]. In addition, cellular senescence may lead to intracellular metabolic dysfunction, further impairing the normal function of the liver. Park et al. showed that the senescence marker protein-30 was associated with the onset and progression of non-alcoholic fatty liver disease (NAFLD), suggesting a close relationship between cellular senescence and NAFLD [132]. Lim et al. reported that the hepatitis C virus core protein improved liver function by inhibiting p16 expression to ameliorate premature senescence [133]. Krizhanovsky et al. found that senescent activated stellate cells limited the progression of hepatic fibrosis, in which NK cells preferentially cleared SnCs, thus facilitating the reversal of hepatic fibrosis [134]. Wiemann et al. showed that hepatocyte senescence is a general sign of cirrhosis, and the formation of fibrosis scars at the stage of cirrhosis may be the result of hepatocyte senescence [135]. Yoshimoto et al. reported that the SASP phenotype in hepatocytes and stellate cells promotes the development of hepatocellular carcinoma (HCC) through the secretion of various tumor-promoting factors [136]. Taken together, these studies suggest that cellular senescence is receiving increasing attention in the prevention and treatment of various liver diseases (Fig. 1).

## 4.Senolytics: Targeted Elimination of Senescent Cells

Senolytics are a class of compounds that selectively target and eliminate SnCs, specifically recognizing and inducing apoptosis of SnCs. It has been demonstrated that senolytics, as a novel anti-aging drug, remove SnCs by targeting specific pathways and molecules, reduce inflammation, enhance tissue function, and thereby alleviate a range of age-related symptoms and prolong lifespan, which is associated with specific pathways and molecules being more active in SnCs than in healthy cells [27, 43]. In the past few years, great progress has been made in the discovery and development of anti-aging compounds and methods, some of which have entered clinical trials for the treatment of age-related diseases. Current reports on senolytics fall into the following categories: (1) small molecules such as dasatinib and quercetin, navitoclax; (2) natural compounds such as fisetin and resveratrol; (3) peptides such as FOXO4-DRI; (4) gene therapy; and (5) nanoparticle-based delivery drugs.

### 4.1 Small moleculars

### 4.1.1 Dasatinib & quercetin (D&Q)

Dasatinib is a tyrosine kinase inhibitor used in the treatment of cancer that clears senescent fat cells [137]. Quercetin is a flavonoid that exhibits antioxidant and free radical scavenging activity both in vivo and ex vivo to prevent various age-related diseases [138]. In basic research, there have been numerous findings demonstrating the ability of senolytic therapy consisting of D&Q to selectively induce the death of SnCs. Novais et al. combined use of the D&Q drug via systemic administration was able to prevent the progression of age-dependent intervertebral disc degeneration, attenuate disc degeneration, reduce systemic inflammation, and improve the physical condition of mice during aging [139]. Saccon et al.’s combined use of the anti-aging drug D&Q reduced the burden of intestinal SnCs and hyperinflammation in mice while altering specific microbiota characteristics [140]. Dungan et al. found that D&Q significantly lowered fasting blood glucose in older mice and enhanced muscle regeneration in older mice after removal of SnCs, yet inhibited muscle regeneration in younger mice [141]. Luo et al. adopted the senolytic combination of D&Q to detect its impact on the intestine of elderly mice [142]. The study found that D&Q treatment will reduce the expression of p21, p16, and Ki67, trigger the removal of apoptosis cells in villi, and stimulate the production of NO in aging mice [142]. These results indicate that D&Q has the potential to alleviate intestinal senescence in aged mice [142]. Clearing SnCs has also been shown to improve brain function and have a significant effect on alleviating neurodegenerative disease in mouse models [21]. An age-dependent increase in p16INK4a SnCs has been found, which is more pronounced in microglia and oligodendrocyte progenitors [143]. Ogrodnik et al. showed a decrease in microglial cell activation and were able to induce a decrease in the expression of p16INK4a and SASP factor in the microglial cell populations, significantly improving cognitive function in aged mice when treated with D&Q [143]. Aged healing tissue SnCs inhibit the growth and proliferation of healing tissue-derived mesenchymal progenitor cells and express high levels of TGF-β1, and fracture induces significant senescence of osteoclasts in aged mice, which inhibits mesenchymal progenitor cells through the expression of TGF-β1, whereas short-term use of D&Q reduces osteoclast SnCs and accelerates fracture healing in aged mice [144]. It has been reported that the combined application of D&Q can reduce the increase of age-related β-galactosidase, reduce the expression of p16 and p21 genes in the white adipose tissue around the gonads, reduce inflammation in the old adipose tissue, and improve systemic metabolic function [145].

Clinically, D&Q has been used for anti-aging treatment and has played an effective role in a variety of age-related diseases [146]. In patients with AD, SnCs can be cleared using D&Q [147, 148]. Idiopathic pulmonary fibrosis (IPF) is a chronic, irreversible age-related pulmonary fibrosis disease that is strongly associated with an increased load of SnCs. Some studies have demonstrated the use of D&Q for the treatment of IPF through an anti-aging combination [149]. In addition to the efficacy of age-related diseases, it also has an obvious role in other chronic diseases. In a study using D&Q in patients with diabetic kidney disease, it was reported that D&Q reduced adipose tissue SnCs, cells with senescence-associated β-galactosidase activity, and adipocyte progenitors with limited replicative potential [148]. The number of adipose tissue macrophages and coronary structures simultaneously attracted, anchored, and activated by SnCs decreased [148].

### 4.1.2 Navitoclax

Navitoclax (also known as ABT-263) is an orally available small-molecule compound that belongs to a class of drugs known as "inhibitors of the Bcl-2 family," a group of proteins that regulate apoptosis and play an important role in the balance between cell survival and death [150, 151]. Among them, Bcl-2 is an apoptosis-inhibiting protein that prevents cells from entering the apoptotic program, leading to abnormal cell survival. Navitoclax, a new senolytic drug discovered in recent years, can specifically inhibit the Bcl-2, Bcl-xL, and Bcl-w proteins in the anti-apoptotic protein Bcl family and activate the caspase signaling pathway to induce apoptosis [68, 152, 153]. In basic experiments, navitoclax was able to effectively eliminate SnCs in vivo and in vitro by targeting key molecules for SnC survival [153]. Chang et al. found that the application of ABT-263 could selectively remove senescent stem cells in the blood system of prematurely aging mice after whole-body irradiation by inducing apoptosis, thereby enhancing the activity of remaining stem cells and restoring the vitality of the hematopoietic and muscular systems of aging mice [153]. Another study reported that ABT-263 was able to target senescent renal tubular epithelial cells and ameliorate chronic kidney disease in mice by removing renal tubular SnCs [154]. The accumulation of SnCs in cartilage can lead to SASP and age-related inflammation and dysfunction. It has been reported that ABT-263 reduces the expression of inflammatory cytokines in osteoarthritic chondrocytes and promotes cartilage matrix aggregation by inducing apoptosis of SnCs, and thus ABT-263 may have a protective effect on the development of post-traumatic osteoarthritis [155]. However, some studies have also reported potential damage to navitoclax on osteocytes [156], and further studies are needed to confirm this view. Doxorubicin is an anticancer drug but has toxic side effects on the heart, and some studies have found that navitoclax eliminates SnCs, significantly reduces markers of senescence and cardiotoxicity, and restores cardiac function in mice with doxorubicin-induced injury [157, 158]. One clinical study demonstrated that navitoclax can be used in combination with cisplatin in patients with head and neck tumors to eliminate senescent tumor cells and slow tumor growth [159].

### 4.2 Natural Compounds

### 4.2.1 Fis*etin*

Fisetin (chemical name: 3,3',4', 7-tetrahydroxy flavone) is a naturally occurring flavonoid that belongs to a class of flavonoids found in plants. Fisetin can induce SnCs to enter an apoptotic state through a variety of pathways and can inhibit Bcl-2 family members, such as Bcl-xL, as well as HIF-α and other components of the SCAP network [160]. Fisetin has been reported as an effective anti-aging agent capable of prolonging life by reducing peroxide levels and enhancing antioxidant cellular responses. Studies have shown that acute or intermittent treatment of fisetin in both early and old mice can reduce aging markers in multiple tissues, reduce inflammation and oxidative stress, improve tissue homeostasis, and reduce a variety of age-related pathological changes [160]. In vivo studies found that the number of senescent renal tubular epithelial cells and myofibroblasts was significantly reduced after fisetin treatment, thereby alleviating renal fibrosis and reducing SASP expression [161]. A recent study found that fisetin could ameliorate skin aging by selectively removing senescent dermal fibroblasts and inhibiting SASP, suggesting that fisetin may be an effective novel therapeutic agent against skin aging [162].

### 4.2.2 Resveratrol

Resveratrol is a potential senolytic compound that exists naturally in some plants. It belongs to a class of polyphenols called flavonoids, which have anti-oxidation, anti-inflammation, anti-cancer, anti-aging, and other effects. Among the anti-aging effects, resveratrol is an activator of the protein deacetylase sirtuin1 (SIRT1), which activates the receptor γ coactivator-1 via peroxisome proliferators [163] and regulates aging through metabolism under nutritional control [164]. Resveratrol promotes the proliferation of human umbilical cord-derived MSCs in a dose-responsive manner, slows down senescence, and induces the expression of SIRT1 while inhibiting the expression of p53 and p16 [165]. Meanwhile, it has been shown that resveratrol protects against ischemia-induced cardiac injury in mice and hypoxia-induced cardiomyocyte injury in rats in vivo and in vitro by modulating SIRT1/p53-mediated cellular senescence and inhibiting NLRP3-mediated inflammatory vesicle activation [166]. Another study found that resveratrol could delay oocyte senescence and enhance developmental capacity by activating mitochondrial autophagy, suggesting that resveratrol-induced mitochondrial autophagy is a potential mechanism to protect against postovulatory egg senescence [167]. Induction of cellular senescence is a novel strategy to inhibit the abnormal proliferation of cancer cells. One study found that resveratrol inhibited tumor cell proliferation and induced tumor cell senescence while increasing SA-β-gal activity and the age-related molecular marker p38MAPK [168]. Resveratrol activates the Nrf2 and SIRT1 signaling pathways, attenuates oxidative stress and mitochondrial dysfunction, and improves renal function, proteinuria, histological alterations, and inflammation in aging mice [169]. It has been shown that resveratrol effectively inhibits the properties of lung cancer stem cells, down-regulates the Wnt/β-catenin signaling pathway, reduces the level of IL-6, and inhibits the development of lung cancer through multi-targeting [170]. Ji et al. demonstrated that resveratrol targeted the tumor suppressor gene DLC1 through the FOXO3a/NF-κB signaling pathway induced by SIRT1, thus triggering tumor cell senescence [168]. Yan et al. found that resveratrol also induced cancer cell senescence by activating endoplasmic reticulum stress through the SIRT1/p38MAPK and NO/DLC1 pathways [171]. In breast cancer and liver cancer cells, resveratrol inhibits cell viability and clonal formation, promotes cell aging, increases SA-β-gal activity, and regulates age-related molecular markers p53, p21, and Lamin B proteins [172]. In a diabetes model, resveratrol ameliorated ethanol-induced pancreatic β-cell senescence by inhibiting the p38MAPK/p16 pathway via a NAD^+^/SIRT1-dependent pathway [173].

### 4.3 Peptides

FOXO4-DRI is an experimental compound used to study the cell biology associated with aging and is a synthetic peptide targeting the FOXO4 protein. FOXO4-DRI selectively induces apoptosis in targeted SnCs by competing for binding to the normally anti-apoptotic FOXO4-p53 [174]. This study also reported that FOXO4-DRI can restore physical fitness, fur density, and renal function in rapidly aging Xpd^TTD/TTD^ mice and naturally aging mice [174]. The p53-dependent pathway plays a role during stress, leading to cell cycle arrest [175]. FOXO4-DRI may play a role by competitively binding p53 with FOXO4, changing the subcellular distribution of p53, thereby activating p53 and leading to cell cycle arrest [176]. By interfering with the interaction between FOXO4 and p53, FOXO4-DRI can selectively induce nuclear rejection and apoptosis of p53 in aging testicular interstitial cells [177]. In naturally aging mice, FOXO4-DRI can effectively improve the testicular microenvironment and reduce age-related testosterone secretion deficiency, which revealed the therapeutic potential of FOXO4-DRI for late-onset male hypogonadism [177]. In vitro studies have found that FOXO4-DRI can effectively remove senescent chondrocytes [178]. In the mouse model of pulmonary fibrosis, the use of FOXO4-DRI can reduce the pathological changes and collagen deposition in mice, and it is revealed that FOXO4-DRI resets the distribution of p53 in the nucleus and reduces the content of total extracellular matrix protein at the same time [179], which is consistent with Baar's study. FOXO4-DRI selectively induces p53 nuclear rejection and eliminates SnCs [174].

### 4.4 Gene therapies

The core of the concept of gene-targeted senescent cell therapy is to delay or reverse the aging process by precisely regulating or eliminating cells that express signature proteins of aging, such as p16INK4a, through specific gene intervention. This strategy is unique in that it is highly precise and is designed to minimize the impact on normal cells, thereby improving the safety and effectiveness of the treatment. This not only opens up new possibilities for the treatment of age-related diseases, but also opens up new avenues for a wider range of anti-aging therapies. p16INK4a is a cyclin-dependent kinase inhibitor that plays an important role in the regulation of cellular senescence. Roshni et al. found that gene knockdown of p16INK4a may play an important role in inducing the antioxidant effect of aging human cardiac stem/progenitor cells and extending lifespan [180]. Khatiwala et al. knocked down p16INK4a using a lentiviral plasmid containing short hairpin RNA (shRNA, an artificial RNA molecule with a tight hairpin turn that silences the expression of target genes by RNA interference) aimed at restoring senescent human cardiac progenitors by upregulating antioxidant and NF-κB signaling pathways [181]. In addition, Wu et al. found that the SASP gene itself and the biomarker of atherosclerosis, IL-1β, induced senescence in human endothelial cells by up-regulating CUX1 and/or down-regulating SATB2, and CUX1 was found to repress SATB2 expression at the transcriptional level [182]. Therefore, upregulation of CUX1 inhibits the expression of SATB2, thereby enhancing CUX1 binding to rs1537371 and subsequently regulating the expression of p16INK4a [182]. Zhang et al. generated RAP1-deficient human embryonic stem cells using CRISPR/Cas9 technology and obtained RAP1-deficient human MSCs and neural stem cells after further directed differentiation [183]. It was also found that RAP1 not only negatively regulates telomere length but also serves as a transcriptional regulator of RELN by adjusting the methylation status of the RELN gene promoter, and that its deficiency enhances the self-renewal capacity of human MSCs and delays aging [183]. LPS can induce senescence in macrophages exhibiting SASP expression and DNA damage. Bromodomain-containing protein 4 (BRD4), a chromosome-binding protein associated with gene expression, plays a key role in the pathological process of breast cancer [184]. Wang et al. showed that an inhibitor of BRD4 could prevent LPS-induced senescence and lipid accumulation in senescent macrophages by decreasing SASP expression in senescent macrophages [184]. Aging causes a chronic pro-inflammatory environment in the hypothalamus, leading to decreased gonadotrophin-releasing hormone secretion and impairing kisspeptin neuron function. Franco et al. found that insulin-like growth factor 1 gene therapy induced kisspeptin production and gonadotropin-releasing hormone release and altered microglia numbers and responsiveness, and this gene therapy may be protective against reproductive decline [185]. Burcak et al. found that NT-3 gene therapy could improve the function of C57BL/6-aged mice and alleviate age-related changes in muscle, bone, and skin [186]. Sadanori et al.’s administration of an adeno-associated viral vector encoding the human DOK7 gene inhibited degeneration of motor nerve endings and muscle atrophy at the neuromuscular junction in a mouse model of amyotrophic lateral sclerosis (ALS) [187]. Hutchinson-Gilford progeria syndrome (HGPS), which is based on mutations in the LMNA gene and the gene encoding the interacting protein, is one of the most serious diseases in laminopathies [188]. LMNA encodes two major alternate splicing transcripts, producing laminin A and laminin C [188]. In the face of the multiple and complex pathogenesis of HGPS, gene therapy seems to be a more suitable method for development. For gene therapy of HGPS, microRNA can silence the specific epigenetic properties of laminin A and premature aging protein transcripts, thus allowing neurons to function normally away from the disease [189-191]. Cytomegalovirus is an effective and safe gene delivery method as a carrier of telomerase reverse transcriptase (TERT) and follistatin (FST) [192]. Dabbu et al. found that mouse cytomegalovirus exogenous to TERT or FST significantly prolonged the lifespan of mice [192]. In a mouse diabetes model, after transferring adenoviral vectors carrying the Sonic Hedgehog gene into the submandibular gland of mice, Hai et al. found that the Sonic Hedgehog gene transfer upregulates the expression of DNA repair-related genes survivin and miR-21 and inhibits the expression of the pro-senescence gene GDF15 downstream of miR-21, thereby promoting DNA repair, reducing oxidative stress, and inhibiting cellular senescence [193]. Ana et al. found that 3-deazaadenosine inhibited an S-adenosyl homocysteinase and indirectly led to the decrease of H3K36me3, which prevented the expression of aging process components [194]. The Lmna^f/f^;TC mice with progerin expression induced by Tie2-Cre exhibit defective microvasculature and neovascularization, accelerated aging, and a shortened life span [195]. Vascular endothelium-targeted SIRT7 gene therapy, driven by an identified intercellular adhesion molecule 2 promoter, improves neovascularization, ameliorates aging features, and extends life span in Lmna^f/f^;TC mice [195]. Although still in the early stages of research and experimentation in the treatment of age-related diseases, gene therapy offers an unprecedented and promising new therapeutic strategy. However, there are still many challenges to be faced in this field, including the refinement of the technology, the long-term effects of the treatment, safety, and ethical issues. In-depth studies of these challenges will be the focus of future research to ensure the successful application and widespread roll-out of gene therapy.

### 4.5 Nanoparticle-Based Delivery

Currently, there are some problems in the clinical use of anti-aging drugs to treat cancer, such as poor targeting and the risk of systemic toxicity. Therefore, improving the targeting of anti-aging drugs and enhancing the delivery efficiency of targeted localized drug delivery to improve drug efficacy has become a research direction of concern for many researchers [196, 197]. Nanoparticle-based delivery is a technique that utilizes nanoparticles as carriers to deliver drugs, genes, proteins, or other bioactive molecules to specific cells or tissues. Nanomaterial drug delivery is a novel therapeutic modality that can improve drug targeting, enhance drug therapeutic effects, reduce drug toxicity and side effects by replacing traditional drug transportation, and solve the current problems of difficult or insoluble drugs and low bioavailability. It has been used in the delivery of anti-aging drugs.

**Table 1:** Role of different senolytics on some common SnCs and age-related diseases.

Compounds/Target genes		Target SnCs	Age-related diseases	Effects	Ref
Small moleculars	D&Q	Glial cell	Neurodegenerative disease	Improves cognition in aged mice	[21]
		Mesenchymal progenitor cell	Fracture	Reduces bone scab and accelerates fracture healing in aged mice	[140]
	Navitoclax	Senescent stem cell	-	Rejuvenation of the hematopoietic and muscular systems in aging mice	[149]
		Chondrocyte	Post-traumatic osteoarthritis	Reduces inflammation levels and promotes cartilage matrix aggregation in osteoarthritis	[151]
Natural Compounds	Fisetin	Renal tubular epithelial cell and myofibroblast	Renal fibrosis	Reduces SASP expression and attenuates renal fibrosis	[157]
		Dermal fbroblast	Skin aging	Effectively improves skin aging	[158]
	Resveratrol	Cardiomyocyte	Ischemic heart disease	Ameliorate cardiomyocyte injury induced by ischemia or hypoxia	[162]
		Lung cancer stem cell	Lung cancer	Multi-targeting to inhibit lung cancer progression	[166]
		Pancreatic β cell	Diabetes	Improves pancreatic β cell senescence and slows the progression of diabetes	[169]
Gene therapies	FOXO4-DRI	Chondrocyte	-	Removes senescent chondrocytes and accelerates chondrocyte growth	[174]
		MRC5 cell	Pulmonary fbrosis	Reduces pulmonary fibrosis	[175]
	p16INK4a	hCPC	Cardiomyopathy	Increasing hCPC proliferation and survival	[176, 177]
	RAP1	hMSC	-	Delay aging	[179]
	SIRT7	HEK293 cell	Hutchinson-Gilford progeria	Improvement of neovascularization, aging characteristics and longevity	[191]
Nanoparticle-Based Delivery	Quercetin surface functionalized Fe_3_O_4_ nanoparticle	Fibroblast	-	Anti-aging	[194]
	Antibody-functionalized mesoporous silicananoparticle	Foamy macrophage and endothelial cell	Aorta atherosclerosis	Targeting areas of aging lesions to slow the progression of atherosclerosis	[195]
	Nano-encapsulated navitoclax	4T1 cell	Breast cancer	Slows tumor growth and reduces metastasis	[201]
	Bone affinity peptide (DSS) modified liposomes loaded with quercetin	BMSC	Osteoporosis	Improves osteoporosis in aged mice	[202]

D&Q: Dasatinib&quercetin, MRC5 cell: Human lung fibroblast cell line, hCPC: Human cardiac progenitor cell, hMSC: Human mesenchymal stem cell, HEK293 cell: Human embryonic kidney cell line, 4T1 cell: Breast cancer cell line, BMSC: Bone marrow mesenchymal stem cell.

Natural polyphenols have limited their application as therapeutic agents due to poor water solubility, chemical instability, and low bioavailability. Lewinska et al. prepared quercetin surface-functionalized Fe_3_O_4_ nanoparticles, which were found to have AMPK-promoting activity and were able to reduce the number of SnCs and inhibit the aging-associated pro-inflammatory response, lowering the levels of inflammatory factors (e.g., IL-8 and IFN-β) [198]. Pham et al. synthesized mesoporous silica nanoparticles with hyaluronidase-responsive drug release characteristics [198]. In in vitro experiments, it was found that the nanoparticle was easy to be taken up by cells and could reduce the level of ROS, the oxidation level of high-density lipoprotein, and the production of pro-inflammatory factors such as TNF-α and IL-6, slow down the aging process, and help improve cell vitality [198]. In vivo experiments have also shown that these nanoparticles can successfully target the areas of aging lesions, deliver the anti-aging drug rosuvastatin to aging atherosclerotic plaques, and alleviate the progression of atherosclerosis in ApoE-/- mice [199]. It has also been shown that exosomes secreted by stem cells from human exfoliated deciduous teeth (SHED-Exos) are rich in anti-aging signals, and SHED-Exos reverses AT-SC aging by regulating histone methylation and inhibiting NF-κB [199]. Systemic administration of SHED-Exos bio-nanoparticles delayed tendon degeneration in a naturally aging mouse model. Interestingly, local delivery of SHED-Exos reduced SnCs and ectopic bone formation and functionally and structurally improved endogenous tendon regeneration and repair in aged rats [200]. Li et al. prepared chiral Cu_x_Co_y_S nanoparticles to selectively induce SnCs apoptosis by using alternating magnetic field and near-infrared (NIR) light irradiation, effectively eliminating SnCs without damaging the activity of normal cells [201]. Nanoparticle drug delivery systems overcome the challenges of poor water solubility and other side effects of drugs, and also show strong application prospects in cataract treatment. A nanosystem of gold nanoparticles containing resveratrol (RGNPs) has been designed as an anti-aging agent to delay the occurrence of cataracts. The experimental results of in vitro studies revealed that spherical RGNPs could delay oxidative stress-induced cellular senescence by decreasing the protein levels of p16 and p21, the ratio of Bax/Bcl-2, and SASP, and also attenuate the lens opacity in sodium selenite-induced cataract rats [202]. Lee et al. found that a locally delivered thrombolytic drug (ABT263-PLGA) based on biodegradable polylactic acid-hydroxyacetic acid copolymer nanoparticles successfully cleared stress-induced SnCs in ischemia-reperfusion-injured rat hearts without systemic toxicity [197]. In other studies, DNA-mediated nanotetrahedral structure particles with NIR responsiveness were constructed, and in vivo experiments showed that the nanoparticles could improve renal function, tissue homeostatic state, fur density and movement ability of aging mouse models under the action of NIR [203]. 4N1Ks peptide is a polypeptide composed of 10 amino acids derived from TSP1 protein, which can target the CD47 receptor on the surface of SnCs and shows anti-aging activity [204]. One study used a biodegradable and biocompatible sphingomyelin nanosystem to modify 4N1Ks peptide to target SnCs [204]. Galiana et al. used nanoencapsulated Navitoclax, a combination therapy that selectively removes SnCs, and the strategy was able to retard tumor growth and reduce metastasis in a mouse model of breast cancer [205]. In one study, liposome loaded quercetin modified with bone affinity peptide (DSS) was constructed. This nanoparticle can effectively clear the SnCs in bone through bone targeting and improve senile osteoporosis in elderly mice [206] (Table 1).

## 5. Senomorphic Strategies: Modulating Senescent Cell Behavior

Although Senolytic is effective in killing SnCs, it is not the only way to combat aging. As an alternative way to intervene in aging, senomorphic therapy does not eliminate SnCs but rather modulates the aging phenotype by enhancing cellular function or inhibiting pathways associated with the expression of SASP components [27]. There are two ways for senomorphism to intervene in aging. On the one hand, it can activate cellular defense and protection mechanisms by inducing mild oxidative stress [207]. On the other hand, senomorphism can inhibit the propagation of senescence by suppressing the expression of SASP-related components and pathways. Some of the common senomorphic agents are NAD^+^ boosters, mammalian target of rapamycin (mTOR) inhibitors, metformin, and sirtuin activators. In contrast to the intermittent dosing of senolytics, senomorphics need to be taken regularly over a long period of time to show effect [208].

### 5.1 NAD^+^ boosters

NAD^+^, fully known as nicotinamide adenine dinucleotide, is involved in cellular respiration and promotes energy metabolism in cells. NAD^+^ is not only an important raw material for the DNA repair system but also a key liaison factor between the nucleus and mitochondria. To a large extent, aging is caused by the lack of NAD^+^ in the human body, and during aging, the NAD^+^/NADH ratio decreases, which in turn causes various aging symptoms [209-211]. It has been shown that increasing the level of NAD^+^ in the organism reduces ROS production, improves mitochondrial function, and increases the detoxification of ROS, thereby decreasing the level of ROS and decreasing the accumulation of damaged mitochondria in cells and animal models of premature aging diseases [212-215]. Why is NAD^+^ negatively associated with age? Current studies believe that one of the important reasons is that with age, NAD^+^ synthesis decreases and consumption increases in the body, so the total amount of NAD^+^ is decreasing [216]. Fundamentally, the factors controlling the synthesis and consumption of NAD^+^ mainly involve two aspects of enzyme activity. On the one hand, the key rate-limiting enzyme (NAMPT) in the NAD^+^ remedial synthesis pathway is involved. On the other hand, NAD^+^-consuming enzymes, namely NAD^+^ glycohydrolase (CD38, CD157, and SARM1), the long-lived protein sirtuin deacetylase family (SIRTs), and poly (ADP-ribose) polymerases (PARPs), are involved [217]. The deficiency of NAD^+^ has become a significant problem in aging and age-related diseases. However, NAD^+^ is a phosphorylated molecule, and due to its high molecular weight and unstable structure, the body cannot absorb and utilize NAD^+^ directly, which has high side effects. It has been proposed that "NAD^+^ boosting," that is, the supplementation of cellular NAD^+^, is a promising treatment for aging and other diseases associated with decreased NAD^+^ concentrations, so NAD^+^ boosters have entered the field of vision [211]. In the following, we will explore the use of "NAD^+^ boosters" in age-related diseases and look forward to further exploring their therapeutic potential.

There are three pathways by which NAD^+^ can be synthesized: (1) Dietary tryptophan is synthesized de novo via the kynurenine pathway. (2) It is generated via the Preiss-Handler pathway using nicotinic acid (NA) as a precursor. (3) It is synthesized via the recycling pathway through nicotinamide or nicotinamide riboside (NR) [218]. Therefore, the supplementation of NAD^+^ precursors has become a hot topic discussed by researchers in recent years on anti-aging. Although NA is a precursor of NAD^+^, there are some limitations on intake and side effects of skin heating and redness, itchiness, or stingling, and hepatotoxicity can be triggered by prolonged overconsumption. Currently, the focus of researchers on NAD^+^ precursors is mainly on NR and nicotinamide mononucleotide (NMN), which have shown some preventive and therapeutic effects and improved age-related pathophysiology and disease conditions.

### 5.1.1 NA

NA is an important B vitamin, named niacin because it was extracted from tobacco in early studies, which plays an important role in human health. In 1915, Conrad Elvehjem discovered that NA could bring relief to dogs suffering from black tongues. In 1929, the correct structure of NA was determined by the English chemist R. D. Compton. Since then, scientists have gradually discovered the important role of NA in the human body. Since then, research on the isolation, structure, and physiological role of NA has been extensively carried out, which makes the application field of NA more and more extensive. NA has been shown to significantly improve cognition and sleep in patients with PD without significant side effects [219]. In 2020, the clinical study of NA as a precursor of NAD^+^ was first reported, and Pirinen et al. used NA as a precursor of NAD^+^ for the clinical study of mitochondrial myopathy [218]. The subjects took NA daily for up to 10 months (4 months in the control group) [218]. The results showed that NAD^+^ in the blood of the patients taking NA increased [220], but all the subjects had varying degrees of flushing and tingling in the limbs, and some even had flatulence, gastrointestinal irritation, and dry skin [220]. According to the National Institutes of Health's big data statistics, side effects occur when taking NA >30 mg. At doses greater than 1000 mg, NA can cause symptoms such as low blood pressure, extreme fatigue, high blood sugar, nausea, heartburn, abdominal pain, blurred or impaired vision, and fluid buildup in the eyes. Therefore, NA should be adequately evaluated for safety prior to use in order to minimize the occurrence of its side effects. In addition, the process of NA conversion to NAD^+^ is relatively complex, requiring the Preiss-Handler path, and at least three enzymes are required to participate in the successful transformation in tissues, which means that if NAD^+^ is supplemented by NA, a very high dose is needed to achieve it.

### 5.1.2 NR

In 2004, Bieganowski and Brenner found in their study that yeast can synthesize NAD^+^ through the Preiss-Handler pathway using the direct conversion of NR to NMN assisted by nicotinamide ribose kinase (NRK), thus bypassing the need for NAMPT in the remediation pathway [221]. Subsequently, Brenner found two genes encoding NRK, NRK1 and NRK2, in the human genome and reported that both fungi and humans could directly synthesize NAD^+^ using NR [221]. NR can induce mitochondrial unfolded protein response and inhibit protein synthesis to reduce aging by improving mitochondrial function, while it can also reprogram dysfunctional adult stem cells and delay the aging of neural stem cells and melanocyte stem cells [222]. Supplementation of NR to increase NAD^+^ storage to maintain healthy mitochondria has the potential to protect neural stem cells and muscle adult stem cell populations from senescence [222]. NR also has the potential to improve the rehabilitation of patients with hematological system failure (including after chemotherapy and radiotherapy). NR can not only improve the functions of hematopoietic stem cells (HSCs) and progenitor cells and promote the production of white blood cells in immunodeficient mice, but also significantly improve the recycling efficiency and regeneration of mitochondria of purified mouse or human hematopoietic stem cells after NR treatment, reduce the toxicity of mouse bone marrow transplantation, and further improve the survival rate of mice after transplantation of HSCs [222]. This also indicates that NAD^+^ supplements such as NR have a promising therapeutic prospect for diseases such as refractory autoimmune idiopathic thrombocytopenic purpura or other autoimmune bone marrow failure syndroms [223]. Oral supplementation of NR molecules has been shown to increase NAD^+^ levels in the heart, liver, and skeletal muscle, as well as oxidative metabolism in high-fat diet-induced obesity. However, the efficacy of orally administered NR is limited because it is poorly targeted in target organs, has low bioavailability, and is subject to rapid degradation in the blood circulation. Therefore, some researchers have proposed to combine nanomaterials with NR to improve NR bioavailability [223]. Nie et al. synthesized NR/RESms/alginate nanopreparations that could increase NAD^+^ levels in serum and multiple organs in mice, which improved the oral bioavailability and efficacy of NR and protected the heart from acute ischemia/reperfusion injury [224]. Therefore, NR is expected to be a potentially effective drug for aging and age-related diseases.

### 5.1.3 NMN

NMN is synthesized from nicotinamide and 5’-phosphoribosyl-1-pyrophosphate by the rate-limiting NAD^+^ biosynthetase NAMPT in mammals, is also synthesized by NR through NRK-mediated phosphorylation, and can be converted to NAD^+^ in humans [211]. NMN is effective in alleviating age-related physiological decline in mice and is able to inhibit age-related weight gain, enhance energy metabolism, improve insulin sensitivity and lipid levels, as well as improve ocular function and other pathophysiology without any toxic side effects [225]. In a clinical study, NMN supplementation in overweight or obese postmenopausal prediabetic women was found to have beneficial effects on their metabolic function, such as increased muscle insulin sensitivity, insulin signaling, and muscle remodeling [226]. Another study showed that the expression characteristics of neurovascular genes in aged mice treated with NMN were compared with those of untreated young mice and aged control mice, and 204 genes that were differentially expressed in the aged neurovascular unit were determined to return to the expression levels of youth after NMN treatment [227]. This suggests that NMN supplementation can promote neurovascular recovery in elderly mice [227]. In addition, NMN is of great help in the treatment of neurodegenerative diseases. Lu et al. found that NMN could significantly reduce the decrease in PC12 cell survival induced by rotenone, and NMN could also significantly reduce rotenone-induced apoptosis and restore intracellular NAD^+^ and ATP levels after rotenone-treated PC12 cells [228]. It can be concluded that NMN can alleviate pathological changes in PD by alleviating apoptosis and improving the energy metabolism of cells [228]. Currently, NMN-related products have been popularized as anti-aging agents in the markets of some countries.

### 5.2 mTOR inhibitors

mTOR is a serine/threonine protein kinase belonging to the phosphatidylinositol 3-kinase (PI3K)-associated kinase family, which plays a key role in the regulation of energy metabolism [229]. As an important part of the PI3K/protein kinase B (AKT)/mTOR signaling pathway, mTOR can recruit other proteins and active factors and assemble into two types of mTOR complexes, which exist in cells in the form of mTORC1 and mTORC2 complexes [230]. It affects protein and lipid synthesis, autophagy, and ubiquitin-proteasome systems and is involved in cell growth, proliferation, metabolism, and survival [230]. Sustained over-activated mTOR signaling will lead to increased levels of cellular metabolism, sustained growth and proliferation, prolonged cellular lifespan, and even cellular immortalization, which can directly or indirectly induce cancer, metabolic, and age-related disorders, whereas inhibition of this state can effectively delay or treat cancers, cardiovascular injuries, and other disorders caused by mTOR over-activation [231]. Various studies have found that various diseases, such as cancer, obesity, type II diabetes, muscle diseases, and neurodegenerative disorders, are associated with abnormal expression or dysfunction of mTOR [230]. Therefore, mTOR may be an effective target for the treatment of a variety of diseases. Nowadays, there are three generations of mTOR inhibitors according to the common site of binding to mTOR: the first generation is mainly rapamycin and its derivatives; the second generation is mTOR kinase inhibitors based on small-molecule ATP-competitive inhibitors; and the third generation is a combination of the first two generations called "Rapalink-1."

### 5.2.1 Rapamycin and its derivatives

Rapamycin, a macrolide produced by Streptomyces hygroscopicus, was first isolated as an antibiotic in 1975 from the soil bacterium Streptomyces hygroscopicus found in the Easter Islands and was named Rapamycin after the Easter Island name "Rapa Nui" [232]. The inhibition of mTOR by rapamycin can selectively inhibit mTORC1 and not mTORC2, but long-term application of rapamycin can reduce the activity of mTORC2, inhibit the secretion of inflammatory cytokines (components of SASP) by SnCs, and selectively inhibit the translation of the membrane-binding cytokine IL1A [233]. Lowering IL1A levels reduces the transcriptional activity of NF-κB, which controls most of SASP [233]. In 2009, Harrison et al. showed that feeding rapamycin extended the maximum lifespan of male and female genetically heterozygous mice by 9% and 14%, respectively, which was the first evidence that lifespan in mammals could be significantly extended by drugs [234]. It is also becoming recognized that interventions need to be started early in life before major age-related senescence occurs and that interventions can sustain life. The journal Science selected this study as one of the major scientific breakthroughs in aging in 2009 [234, 235]. Salvatore Oddo et al. discovered that rapamycin prevented cognitive loss in transgenic AD mice, and since then, research on the role of rapamycin in anti-aging has increased dramatically [236]. It has also been shown that rapamycin prevents Aβ1-42-induced inhibition of γ-oscillations [237], which to some extent also plays a role in inhibiting the development of AD. Some mouse studies have shown that it has powerful pharmacological effects in delaying aging, prolonging life, and alleviating age-related diseases [238, 239]. Mice treated with rapamycin lived longer, had stronger hearts, and responded better to vaccination [240]. Only the Bitto et al. study reported no effect of rapamycin on lifespan [241]. At high intraperitoneally administered doses (8 mg/kg/day), rapamycin had no effect on the lifespan of female mice but increased the lifespan of male mice by 61%, while it was also found that dietary administration of rapamycin extended the lifespan of female and male mice by 39% and 45%, respectively. As far as is known, rapamycin is effective in animal experiments in a large dose range, and even at high doses, there is no significant effect on animal lifespan. It is also effective in both sexes of mice, except that female mice will be more sensitive to rapamycin, but at high doses, the difference is not significant [241, 242]. Johnson et al. showed that rapamycin prolonged the lifespan of Ndufs4-/- mice, especially at ultra-high doses of rapamycin, which were 28-fold higher than the dose of rapamycin initially shown to prolong the lifespan of mice in the Harrison et al. study [234, 243]. Reifsnyder et al. studied C57BLKS/J-Lepr^db^ mice, a mouse model of type 2 diabetes, and found that rapamycin improved the kidney and heart function of female C57BLKS/J-Lepr^db^ mice and doubled the lifespan of female mice, but had no effect on the lifespan of male mice [244]. These studies all suggest that rapamycin may be an ideal anti-aging supplement. However, rapamycin still has certain defects, such as poor water solubility and stability and low bioavailability. More seriously, rapamycin can cause a series of dose-dependent side effects, such as immunosuppression, hyperglycemia, dyslipidemia, and interstitial pneumonia [245]. Therefore, in order to solve these problems, many researchers carried out a series of structural modifications to rapamycin [246]. As a result, a series of rapamycin derivatives, such as everolimus and temsirolimus, have emerged and have been used in clinical practice. Compared to rapamycin, everolimus exhibits higher bioavailability, a shorter terminal half-life, and different blood metabolite patterns [246].

Currently, Everolimus is the only mTOR inhibitor approved by the US FDA (Food and Drug Administration). Evolimus binds to the intracellular protein FKBP12 to form the inhibitory complex mTORC1, which can inhibit the activity of mTOR. Inhibition of the mTOR signaling pathway can lead to decreased activity of S6K1 and 4E-BP1, thus interfering with the translation and synthesis of proteins related to the cell cycle, angiogenesis, and glycolysis [247]. Everolimus has antitumor efficacy and is used in the treatment of breast cancer and advanced renal cell carcinoma, as well as some neuroendocrine tumors, often in combination with exemestane [248-250]. In cardiovascular disease, everolimus can lower blood pressure and reduce organ damage [251]. It has also been shown that everolimus does not affect the histopathological signs of LPS or LPS/oleic acid-induced lung injury and also exerts some immunomodulatory effects in injury models [252]. Everolimus has antiproliferative effects in cancer cell lines and animal models and inhibits mTOR well [253, 254]. It is well known that mTOR is upregulated in most HCC cells [247]. Subsequent phase I and II studies also found that everolimus was well tolerated and delayed disease progression in patients with advanced HCC [255]. Yamamoto et al. screened anti-aging drugs that selectively kill SnCs and found that the combination of anti-apoptotic Bcl-2 family protein inhibitor navitoclax with evolimus and gemcitabine could increase the number of cleaved caspase-3-positive apoptotic cells, effectively reducing the number of surviving cells in malignant meningioma and tumor size [256]. It was also found that everolimus, in combination with gemcitabine, inhibited tumor growth in vivo significantly more than either treatment alone [256].

### 5.2.2 Small molecule ATP competitive inhibitors

Small-molecule ATP competitive inhibitors can simultaneously target mTOR or mTOR and PI3K, including pyrazolopyrimidine, imidazolopyrimidine, pyridinopyrimidine, thienopyrimidine, triazines, benzodiazolidones, quinolines, imidazolidinones, etc. Among pyrazolopyrimidines, pp242 has a dose-dependent effect on cell proliferation [231]. It is more potent than rapamycin in inhibiting cell proliferation at high doses, and the high efficiency of these compounds can be attributed to the similarity of the kinase binding pattern of the pyrazolopyrimidine ring to that of the ATP adenine ring [231]. Studies have shown that pyrazolopyrimidines, quinolines, and pyrimidines have significant inhibitory effects on breast cancer as well as gliomas [201, 257-259]. Researchers are gradually increasing the exploration of second-generation small-molecule ATP-competitive inhibitors in an effort to increase their potency and improve their performance. Krapf et al. synthesized well-characterized inhibitors called 2,4-disubstituted pyrimidine derivatives with low intrinsic cytotoxicity and high potency and selectivity for ATP-binding cassette G2, confirming its ability to reverse multidrug resistance during cancer chemotherapy when co-administered with active metabolites of irinotecan and mitoxantrone [260]. PF3600 is a pyrimidine that effectively inhibits CDK2/4/6 activity and has shown efficacy in a variety of in vivo tumor models. Currently in Phase 1 clinical trials (PF3600), it will provide a new treatment option for cancer patients whose CDK4/6 inhibition is not sufficient to alter disease progression [261]. A study has shown that GDC-0077, a small-molecule inhibitor targeting the PI3K signaling pathway that can be used for the treatment of patients with PIK3CA-mutant breast cancer, is currently in phase III clinical trials [262]. Premkumar et al. found that JSI-124 in combination with dasatinib inhibits growth and induces apoptosis in malignant glioma cells [263]. Yang et al. found that a multi-enzyme complex" pyrimidinosome" can regulate the role of pyrimidine flux and ferroptosis and proposed drug strategies targeting pyrimidine bodies in cancer therapy [264].

### 5.2.3 Rapalink-1

Researchers identified Rapalink-1, a combination product of rapamycin and mTOR inhibitors. mTOR-xenograft mouse models of breast cancer and glioblastoma suggest that Rapalink-1 is more sensitive than rapamycin and AZD8055, but large sample clinical data for this drug have not been reported [231]. In addition, Negayama et al. found that Rapalink-1 combined with hydroxychloroquine may be a potential therapeutic agent for advanced undifferentiated pleurosarcoma [265]. Mice treated with Rapalink-1 showed significantly delayed tumor growth and a significant reduction in CD44^+^ expression in cells collected from tumors of treated animals [266]. Recently, Rapalink-1, a drug linking rapamycin and the mTOR kinase inhibitor MLN0128, was developed with favorable therapeutic efficacy in breast cancer cells harboring mTOR resistance mutations [267]. It can be seen from the above that Rapalink-1, the third-generation mTOR inhibitor, has a good anti-tumor effect and has great application prospects in cancer therapy.

### 5.3 Metformin

Metformin was proven to be effective and safe for diabetes in 1995 and is currently the most widely used first-line drug for the treatment of type 2 diabetes. It has been reported to delay aging by affecting key signature events of aging, including dystrophic perception, loss of protein stability, mitochondrial dysfunction, altered cellular communication, telomere wear, genomic instability, etc. It also plays a protective role in reducing the progression of various age-related diseases [268]. Metformin has the effect of repairing DNA damage, scavenging ROS, protecting cells, and exerting anti-photoaging effects by inhibiting mitochondrial autophagy and the PI3K/AKT/mTOR signaling pathway [269]. Metformin can reduce the production of non-mitochondrial ROS in SnCs by inhibiting NADPH oxidase 4, which may improve the quality of life of patients with cancer caused by long-term exposure to radiation through short-term adjuvant anti-aging intervention [270]. In a high glucose-induced senescent cell model, autophagy and SIRT1 activity were inhibited in human lens epithelial cells, and the use of metformin could mediate SIRT1 inhibition of cellular senescence [271].

Metformin has been shown to be effective against age-related diseases such as degenerative bone diseases, cardiovascular diseases, neurodegenerative diseases, tumors, obesity, and other metabolic abnormalities [268]. The results of the study showed metabolic and non-metabolic effects of metformin on aging in the elderly, providing evidence for the anti-aging effects of metformin [272]. Mesenchymal stem cell transplantation is a promising method for the treatment of chronic kidney disease, but chronic kidney disease can cause the senescence of mesenchymal stem cells. Kim et al. found that metformin can target senescent mesenchymal stem cells in chronic kidney disease and effectively reduce inflammation and fibrosis [273]. Metformin counteracted H_2_O_2_-induced senescence by activating the AMPK/mTOR-dependent autophagy pathway, while metformin-pretreated adipose MSCs could potentially inhibit osteoarthritis progression [274]. Metformin may effectively alleviate the aging of lens epithelial cells and the formation of age-related cataracts by inactivating the AMPK pathway and enhancing the autophagy pathway [275]. It has been shown that metformin ameliorates doxorubicin-induced upregulation of senescence markers, reduces the secretion of SASP factors and adhesion molecules, which may be protective against doxorubicin-induced vascular senescence and endothelial dysfunction, and ameliorates infection-induced hyperinflammation in doxorubicin-treated cancer survivors [276]. Meanwhile, metformin can attenuate the damage caused by cancer chemotherapy; for example, it can attenuate the intestinal damage associated with chemotherapy by inhibiting cellular senescence and oxidative stress [277]. In PD treatment, metformin ameliorated the inflammatory response, oxidative stress, and bone loss in PD rats and attenuated oxidative stress-induced senescence by stimulating autophagy, which may be beneficial from an anti-aging perspective [278]. The study has shown that metformin inhibits SnC phosphorylation and aggregation in PD mice, reduces oxidative stress, and improves motor and cognitive dysfunction in MPTP-induced mice [279, 280]. In addition, the activation of the TGF-β pathway and the inactivation of AKT caused by the combination of metformin and simvastatin may be related to the induction of aging, related secretory phenotypes, and dysregulation of spliceosome components, and the combination of metformin and simvastatin can also play a powerful anti-tumor effect in vitro and in vivo experiments [281]. In a recent follow-up study, metformin significantly inhibited the expression of pro-inflammatory cytokines and reduced the associated risk of death in elderly patients with diabetes [282]. In addition, metformin has been shown to effectively reduce the pathogenesis and mortality of cardiovascular diseases [283]. Elderly patients taking metformin have a lower risk of death from cardiovascular disease compared to other hypoglycemic agents [284].

### 5.4 Sirtuin activators

So far, caloric restriction (CR) is the only effective way to extend life without genetic or pharmaceutical intervention [285]. CR decreases glucose, amino acids, and lipids, as well as insulin and growth hormone, and elevates NAD^+^ and ATP levels [285]. In 1960, Berg and Simms proposed that the loss of body fat played a decisive role in mediating the beneficial effects of CR on fertility, age-related diseases, and longevity in rats [286]. Since then, there have been an increasing number of studies on CR and aging and age-related diseases, and more evidence has shown that CR strongly delays age-related diseases (such as chronic kidney failure) while also extending the lifespan of many different species [287-289]. In addition to caloric restriction, genetic screening of long-lived mutants of Saccharomyces cerevisiae showed that the SIRT2 gene could slow down aging in this species [290, 291]. SIRT2 was later identified as a histone-dependent deacetylase required by NAD^+^ to restrict calories to extend the lifespan of yeast [292, 293]. It has also been found that mammalian sirtuins are a family of NAD^+^-dependent enzymes homologous to the Saccharomyces cerevisiae gene SIRT2 [288]. To date, seven sirtuins (SIRT1 to SIRT7) have been identified in humans. The most common reaction catalyzed by human sirtuins is deacetylation, which is mainly mediated by SIRT1-3, and SIRT4-7 also has weak activity. All of these isoforms share an evolutionarily conserved w275 residue catalytic region, while their N- and C-terminal structural domains differ in size and sequence, which affects their substrate preference, catalytic response, and subcellular localization [294]. Sirtuins have been shown to have remarkable ability to prevent disease and even reverse aging by catalyzing protein lysine deacylation and mono-ADP-ribosylation through NADP-dependent catalytic mechanisms [295]. Currently, SIRTs serve as key anti-aging targets, of which SIRT1, SIRT6, and SIRT3 may be viable anti-aging targets for future research [94]. SIRT6 has been a focal point of aging research as progeria-like phenotypes have been associated with SIRT6 deficiency. SIRT6 has multiple molecular functions, including DNA repair and heterochromatin regulation, which makes it a hub for regulating genomic and epigenome stability. Genomic instability caused by persistent DNA damage and accumulated mutations, as well as changes in the epigenetic landscape and suppression of repetitive genetic elements, has become a mechanism that drives aging in organisms [296]. The study has shown that there is a relationship between SIRT6 expression and lifespan, in which the overexpression of SIRT6 prolongs the lifespan of male mice [297]. In this study, SIRT6 overexpression decreased serum levels of insulin-like growth factor 1 and increased the expression of insulin-like growth protein binding protein 1 in male mice, bringing its values closer to those of control female mice [297]. Kawamura et al. showed that in budding tunicate Polyandrocarpa misakiensis, recruitment of SIRT6 by Yin-Yang-1 to the mitochondrial transcription factor A (Tfam) gene via YY1-associated factor 2 resulted in mitochondrial downregulation associated with senescence [298]. In addition, researchers have found that both the SIRT1 and SIRT3 genes contribute to improved healthy aging in mammals [299]. SIRT1 is an attractive regulatory target for anti-aging activity in aging studies because it regulates transcription factors associated with it [300]. SIRT1 is involved in a wide range of age-related processes, such as cell survival, stress adaptation, cell growth and differentiation, and mitochondrial function, by regulating the acetylation state of intracellular substrates [301, 302]. The expression and activity of SIRT1 decrease with age, and enhancement of its activity extends the lifespan of various organisms, including mammals, and ameliorates many age-related diseases. These include cancer [303], metabolic diseases (such as diabetes) [304], cardiovascular diseases [305], neurodegenerative diseases [306], musculoskeletal disorders [307], and kidney diseases [308]. Zhang et al. found that the expression of TGF-β1 was upregulated in SnCs [309], whereas the activation of the TGF-β1/SMAD3 signaling pathway was inhibited in a dose-dependent manner by bergenin and myricetin, and the treatment of these compounds also ameliorated hepatic and pulmonary fibrosis in mice [310-312]. The activation of SIRT1 down-regulates the expression of TGF-β/SMAD2/3, thereby improving the remodeling of the airway, heart, and kidney in mice [313-316]. These results suggest that petroselin and myricetin have the ability to modulate the SIRT1/TGF-β1/SMAD3 pathway and may be promising candidates for anti-aging and treatment of lung remodeling. Ma et al. identified SIRT2 as a molecular target for rejuvenating senile oligodendrocyte progenitor cells (OPCs) [317]. β-NMN delays myelin aging under normal conditions and enhances myelin regeneration in the demyelinated aged CNS by restoring nuclear entry of SIRT2 in aged OPCs [317]. Of the seven sirtuins found in humans, SIRT3 is the only sirtuin for which there is a correlation between polymorphisms and the prolongation of human lifespan. The variable number of tandem repeat polymorphism in intron 5 of the SIRT3 gene has allele-specific enhancer activity. Activation of SIRT3 slows down or prevents mitochondrial dysfunction in response to neurodegenerative disorders, demonstrating potent neuroprotective effects [318]. A growing body of research has demonstrated that SIRT3 has unique roles and potential in the central nervous system, such as in cases of cerebral ischemia [319], traumatic brain injury [319], tumors [320], and neurodegenerative diseases [321].

**Table 2:** Role of different senomorphics on some common SnCs and age-related diseases.

Compounds		Target SnCs	Age-related diseases	Effects	Ref
NAD^+^ boosters	NA	-	PD	Improves movement, cognition and sleep	[219]
	NR	Senescent stem cell	-	Extends life span of mice	[217]
	NMN	PC12	PD	Attenuates apoptosis and improves energy metabolismPD-like behavior and neuropathology alterations	[223]
		Periskeletal muscle cell	Diabetes	Increases skeletal muscle insulin signaling, insulin sensitivity, and muscle remodeling in overweight or obese pre-diabetic postmenopausal women	[221]
mTOR inhibitors	Rapamycin	Cardiomyocyte, vascular endothelial cell	Heart disease	Reduces plaque and cardiac hypertrophy, infarct size, improves cardiac function	[230, 238]
		Neuron	AD	Improves memory, reduces anxiety and depression, and extends lifespan in AD Mice	[231]
		Pancreatic β-cell	Diabetic, Kidney disease	Increases insulin sensitivity, improves glucose homeostasis, improves renal function, and prolongs life span in mice	[238]
	Small molecule ATP competitive inhibitors	Breast cancer cell, Glioma cell	Breast cancer, Glioma	Induces apoptosis of cancer cells and inhibits proliferation and migration of cancer cell	[262,263]
	Rapalink-1	Renal cell carcinoma cell	Renal cell carcinoma	Good anti-tumor effects	[259]
Metformin		MSC	CKD	Exhibit a therapeutic benefit to target accelerated senescence of CKD MSC.	[265]
		Adipose-derived MSC	OA	Inhibits the progression of OA	[266]
		Dopaminergic neuron	PD	Reduces neuronal apoptosis and improves motor and cognitive impairment in MPTP-induced mice	[270, 271]
Sirtuin activators	Myricetin	Liver cell	Liver fibrosis	Reduces liver fibrosis	[303]
		Cardiomyocyte	Myocardial fibrosis	Reduces myocardial diastolic dysfunction	[304]
		Glomerular mesangial cell	Renal fibrosis	Reduces renal fibrosis	[305]
	Resveratrol	Cervical cancer cell	Cervical cancer	Inhibits the progression of cervical cancer	[330]
		Neuron	Neurodegenerative disease	Reduces neuroinflammation and oxidative stress and inhibits the progression of neurodegenerative diseases	[317]
Senostatics	Nobiletin	PC12 cell, HT22 cell, SK-N-SH cell	AD, PD	Effectively reduces symptoms of AD and PD and prevents progression of cognitive function in patients	[343]

NAD^+^: Nicotinamide adenine dinucleotide, NA: Nicotinic acid, NR: Nicotinamide riboside, NMN: Nicotinamide mononucleotide, PD: Parkinson disease, AD: Alzheimer disease, CKD: Chronic kidney disease, MSC: Mesenchymal stem cell, OA: Osteoarthritis, PC12 cell: Rat adrenal medullary pheochromocytoma cell line, HT22 cell: Hippocampal neuronal cell line, SK-N-SH cell: Human neuroblastoma cell line.

As a major member of sirtuin activators, resveratrol can significantly reduce the secretion of peripheral inflammatory factors in patients with grade I hypertension by improving intercellular communication [322]. Resveratrol is a natural compound that activates SIRT1 and may help treat or prevent obesity, as well as tumor development, age-related decline in heart function, and loss of neurons. The anti-aging mechanism of resveratrol is mainly to improve oxidative stress, relieve inflammation, improve mitochondrial function, and regulate apoptosis. Several studies have shown that resveratrol can prolong the lifespan of Caenorhabditis elegans [323] and Drosophila melanogaster [324]. Resveratrol's role as an immune response modulator has been demonstrated in both in vitro and in vivo studies, reversing immune senescence in older rats, reducing inflammatory responses in rodents, and improving immune activity against cancer cells [325]. Resveratrol has a protective effect against neurodegenerative diseases by enhancing neurotransmitter secretion, increasing new neuron production, reducing neuroinflammation and oxidative stress, and promoting hippocampal neurogenesis [326]. Some scholars have found that resveratrol can induce neuronal autophagy in cerebral ischemia/reperfusion rats and has a protective effect on neurons [327-329]. Resveratrol can also reduce the production of superoxide, increase the level of NO, regulate the activity of the renin-angiotensin system, improve oxidative stress, and restore the activity of SIRT1 to play a cardioprotective role [326]. It has also been shown that resveratrol can target or inhibit certain signaling molecules involved in the proliferation and survival of cervical cancer cells and has significant therapeutic potential in the treatment of cervical cancer [330].

### 5.5 Senostatics

Senostatics targets the underlying processes that lead to aging, does not destroy cells, and is mainly a therapeutic strategy around SASP secreted by SnCs, which belongs to a broad category of antiaging agents. Senostatics can reduce the harmful effects of SnCs by inhibiting aging traits [331]. At present, there are two therapeutic strategies for SASP: one is to regulate the SASP regulatory network in SnCs, and the other is to inhibit specific harmful components produced in SASP. Then how to widely regulate the SASP regulatory network in SnCs. At present, the therapeutic targets of anti-aging agents include NF-κB [332], miR-34a [333], GATA4 [334], mTOR [335], BRD4 [336], cGAS/STING [337], etc. Currently, antioxidants or NF-κB inhibitors can be used as effective anti-aging agents [338, 339]. Additional evidence suggests that a variety of flavonoids, polyphenols, and other phytochemicals may have anti-aging activities [340, 341]. In vitro experiments proved that interventions such as metformin, rapamycin, and other mTOR inhibitors could not eliminate SnCs, but after metformin or rapamycin was used in mice for a short period of time, researchers found that multiple markers of aging levels in mice were significantly lower than before treatment [342]. Nobiletin has been shown to significantly improve cognitive defects in an animal model of AD as well as motor and cognitive deficits in an animal model of PD [343] (Table 2).

## 6. Key Challenges in Senolytic and Senomorphic Therapies

We understand that SASP that eliminates SnCs or regulates SnCs has significant therapeutic potential in aging and aging-related diseases, but the accompanying problems and challenges also need to be considered and addressed. At present, some key points still need special attention, such as the complexity of senescent cell behavior, the specificity of senolytics and senomorphics, long-term safety, drug resistance, and the impact on the body's immune system and organs.

### 6.1 Specificity of Senolytics and Senomorphics

One of the key challenges is ensuring that these compounds specifically target SnCs without affecting normal, healthy cells. Lack of specificity can lead to unintended side effects and may harm normal tissue function. This is a key issue facing anti-aging drugs at present: whether anti-aging drugs that induce all SnCs are excellent treatment drugs. There are some common targets and common pathways between normal cells and SnCs. When anti-senescent drugs play a role in the body, how to avoid affecting normal cells while targeting SnCs, that is, how to make anti-aging drugs more specific to avoid off-target effects [344]. With the development of anti-aging drugs, there may be specific drugs with more targeted tissues or cells in the future. These specific drugs may have higher efficacy, fewer side effects, and greater biosafety than current drugs [147]. Therefore, on the basis of determining the appropriate anti-aging drugs, we should find a more suitable delivery system that can target specific tissues and cells so that it can achieve maximum efficacy and the lowest toxic side effects [345].

### 6.2 Long-term Effects and Safety

Since cellular senescence is a natural part of aging and has roles in processes like wound healing and cancer suppression, the long-term effects of continuously eliminating or altering SnCs are not fully understood. The safety profiles of these treatments need thorough evaluation in long-term studies. Natural anti-aging drugs may have the advantage of low toxicity, but overuse in the body can still cause certain harmful side effects. Senolytics and senomorphics may act on important pathways in the body and have certain toxic and side effects on the body. Long-term weakening of certain aging phenotypes may cause tissue metabolic disorders and then affect the normal metabolism of the body. If it is combined with anti-aging drugs that need long-term medication, it is very likely to cause adverse reactions in the human body, and it is necessary to use caution. Moreover, most natural anti-aging drugs still do not understand their mechanism of action, and their molecular targets have not been identified, so determining their molecular targets and their mechanism of action is extremely critical for the improvement of anti-aging agents in the future [346]. We need to find good therapeutic targets for which anti-aging drugs can target SnCs without affecting the normal function of normal cells. In addition, we can also modify anti-aging drugs with some polymers so that they specifically target specific SnCs, and it is best to facilitate the timely degradation of anti-aging drugs after they play a role to avoid long-term accumulation of drugs in the body, causing excessive toxic side effects. At the same time, intermittent drug administration can also be considered, which has greater feasibility in the human body and also reduces the side effects of the drug to a certain extent [347]. All of these indicate that the long-term efficacy and safety of drugs are also drug use issues that we need to pay more attention to.

### 6.3 Complexity of Senescent Cell Behavior

SnCs have diverse functions and characteristics, depending on their tissue of origin and the senescence-inducing stimuli. This complexity poses a challenge in predicting the outcomes of targeting these cells and in designing universally effective therapies. Therefore, we need to determine when and where anti-aging agents work in order to avoid disrupting the positive effects of aging cells. To achieve this, we need to pay special attention to two issues. First, in the face of the complexity of SnCs, we should further explore the particularity of SnCs in different tissues and the positive and negative effects on tissue function. Many SnCs play a very important role in the body, and excessive removal of these SnCs will cause disorder in the body [345]. For example, clearing old blood vessel endothelial cells is not replaced by nearby younger cells of the same type but causes tissue fibrosis [348]. In addition, we also need to pay attention to whether there are differences in the effects of anti-aging agents on different types of SnCs, so as to avoid the lack of specificity of the treatment of SnCs and then eliminate excessive SnCs or excessive interference with the behavior of SnCs, thereby affecting the body's organs and tissue disorders. For example, the D&Q combination did not eliminate senescent hepatic sinusoidal endothelial cells as originally expected, and D&Q effectively cleared p16^High^ macrophages but not p16^High^ CD31-positive liver sinusoidal endothelial cells and adipocytes [348].

### 6.4 Resistance to Senolytics

Like in cancer therapy, there is a possibility that SnCs could develop resistance to senolytics over time. Understanding and overcoming this resistance is crucial for the efficacy of these treatments. Resistance to antibiotics and anticancer therapies is more likely to develop than resistance to antiaging therapies targeting SnCs, which occurs because, unlike microbes and cancer cells, SnCs do not divide, thus reducing the risk of resistance development [347, 349, 350]. For example, navitoclax targets only some anti-apoptotic members of the Bcl-2 protein family but not the anti-apoptotic protein Mcl-1, which leads to overexpression of the anti-apoptotic protein Mcl-1 and rapid development of drug resistance in cancer cells [351]. It has also been shown that cells exposed to resveratrol become more resistant to hydrogen peroxide and succulentacin after several weeks, so the acquired resistance that resveratrol may develop over the course of long-term treatment should be fully considered in clinical trials [352]. We should use modern science and technology to explore as many gene therapy targets of senolytics as possible and use the multi-omics map of cancer cells to provide technical help for new anti-cancer treatment strategies and to avoid the occurrence of anti-aging drug resistance to a greater extent.

### 6.5 Systemic Effects on Aging and Disease

The systemic impact of removing or altering SnCs on overall aging processes and age-related diseases is not fully understood. It's crucial to study how these interventions affect the entire organism and various age-related pathologies. Eliminating SnCs and influencing the behavior of SnCs has many benefits for the whole body, such as improving the metabolism of organs and tissues throughout the body, reducing systemic inflammation, enhancing tissue regeneration and repair, and prolonging the healthy lifespan of the body. For example, targeting skin senescence through topical use of senolytics and senomorphics may contribute to the development of novel anti-aging strategies to delay the onset of systemic aging and age-related diseases. Although anti-aging drugs have many obvious benefits for the human body, it is also necessary to pay attention to the toxic side effects of excessive use of anti-aging drugs on the body. Studies have shown that ABT-263 or FOXO4-DRI will aggravate the deterioration of lung hemodynamics after eliminating senescence cells [176]. It has also been shown that navitoclax not only lyses and eliminates some SnCs but also inhibits certain types of cells and, more seriously, exhibits severe cytotoxic effects on platelets and neutrophils, leading to thrombocytopenia and neutropenia [344]. Therefore, if long-term administration is required, it is necessary to choose appropriate and mild anti-aging drugs or try to choose local administration and other measures to prevent the side effects of anti-aging drugs on the body.

### 6.6 Immune Response and Inflammation

Modulating the immune system's interaction with SnCs can be double-edged. While it might aid in clearing these cells, it could also trigger excessive immune responses or chronic inflammation, leading to other complications. The lack of specificity of anti-aging drugs for harmful SnCs may lead to extensive clearance of SnCs from the body, which in clinical trials leads to thrombocytopenia and lymphocytopenia and affects the body's immune system [353, 354]. Therefore, precise elimination of SnCs would be critical to mitigating the excessive immune response and inflammation of anti-aging drugs. In addition, while anti-aging agents may improve immune function by targeting the aging immune system, immune-mediated diseases may be further worsened by increased immune activation and inflammation [355]. Therefore, targeting anti-aging drugs to SnCs for the treatment of aging-related diseases should be closely monitored for subsequent abnormalities in the immune system as well as excessive inflammatory responses.

## 7. Combination Therapies

Monotherapy for cancer is often prone to drug resistance and tumor recurrence, which reduces efficacy. Combination drug therapy has been shown to reduce drug resistance while minimizing potential side effects [356]. Since SnCs also develop resistance to most of the currently available senolytic drugs, it is reasonable to use combination drugs for SnCs. Navitoclax can inhibit the expression of the anti-apoptotic proteins Bcl-2, Bcl-xL, and Bcl-w [152]. However, inhibition of Bcl-xL results in thrombocytopenia, leading to clinical applications of navitoclax being toxic [153, 357]. Alejandra et al. demonstrated in a xenograft mouse model of invasive human triple-negative breast cancer that the combination of the senescence-inducing agent pipecitlib and the senescence-solubilizing agent navitoclax synergistically eliminated SnCs and reduced tumor growth and lung metastasis, while reducing the cytotoxicity of navitoclax and minimizing potential side effects [358]. Kohei et al. reported that pyrrole-imidazole polyamide conjugated with the mitochondria-delivering moiety triphenyl-phosphonium with targeted mtDNA mutations has the potential to induce senescence in some tumor cells and also has the potential to induce apoptosis when combined with anti-aging drugs in vivo [359]. Xu et al. reported that pan-mTOR inhibitors enhanced the senescence activity of navitoclax, thereby reducing the accumulation of SnCs and alleviating the senescent phenotype in cells and Drosophila, providing new evidence for the advantages of combinations in anti-aging therapy [360]. Taken together, the combination of senolytics and senomorphic strategies offers a promising avenue for developing interventions that target the complex biology of aging. Research in this area is still evolving, and the synergies between the two approaches have great potential to improve the effectiveness of therapies aimed at promoting healthy aging and treating age-related diseases.

## 8. Immune-Based Approaches

Although senolytic drugs such as ABT-263 and D&Q are new options for the treatment of many diseases, they still lack potency and specificity and are prone to side effects [361]. SnCs are inherently immunogenic and are the target of immunological monitoring [362-364]. Immune cells can promote or inhibit the clearance of SnCs by interacting with them, establishing a chronic pro-senescent and pro-inflammatory environment, thereby recruiting and activating immune cells through SASP, and therapies that enhance the clearance of SnCs by immune cells may help to reduce the burden of SnCs [365]. The aging phenotype varies depending on the environment and triggers, and it is clear that different types of immune systems are involved in the clearance of SnCs, with different immune cell types synergistically clearing SnCs from different tissues [366]. So far, different types of immune system cells are involved in the clearance of SnCs, with macrophages and NK cells playing a crucial role. In particular, macrophages are involved in the clearance of SnCs during embryogenesis [367, 368], as well as the clearance of senescent erythrocytes [369], or precancerous or malignant SnCs [370, 371]. In hepatocellular carcinoma cells, neutrophils have also been shown to be involved in tumor clearance after senescence induction due to the reactivation of p53. On the other hand, NK cells have been described as effective targets of senescent hepatic stellate cells, senescent hepatocellular carcinoma cells after p53 repair, and drug-induced senescent multiple myeloma cells [372-374].

A chimeric antigen receptor T (CAR T) cell is a type of T lymphocyte that has been engineered to express a chimeric receptor that targets a specific antigen, triggering T cell activation and target cell killing, and is commonly used in cancer therapy. Amor et al. discovered that CAR T cells could serve as a new and powerful method for clearing SnCs [375]. Urokinase-type plasminogen activator receptors are up-regulated cell surface proteins in SnCs. Upar-specific CAR T cell therapy can prolong the survival of mice with lung adenocarcinoma induced by the combination of senescence-inducing drugs MEK and CDK4/6 inhibitors, restore tissue homeostasis in mice with chemical or diet-induced liver fibrosis, and improve liver function [375]. Natural killer group 2 member D ligands (NKG2DLs) were found to be up-regulated in SnCs from various tissues in rodents and non-human primates. Therefore, Yang et al. developed a chimeric antigen receptor CAR T cell targeting human NKG2DLs that can effectively and selectively reduce human SnCs induced by carcinogenic stress, replication stress, DNA damage, or P16INK4a overexpression [375]. Moreover, autologous T cells with human NKG2D CAR can effectively clear naturally occurring SnCs in non-human primates without any adverse effects [277]. Despite the significant positive effects of CAR T cells on cancer and liver fibrosis, there are still some safety concerns with the use of CAR T cells, such as the antigen being highly expressed in SnCs, but it can also be expressed by non-SnCs, and its low level, but still causing side effects [376]. In addition, CAR T cell-mediated cell death may trigger cytokine release syndrome [377]. Therefore, how to improve the clinical transformation of CAR T and pay attention to clinical safety and efficacy are the current research difficulties to be solved [378].

**Figure 2.**
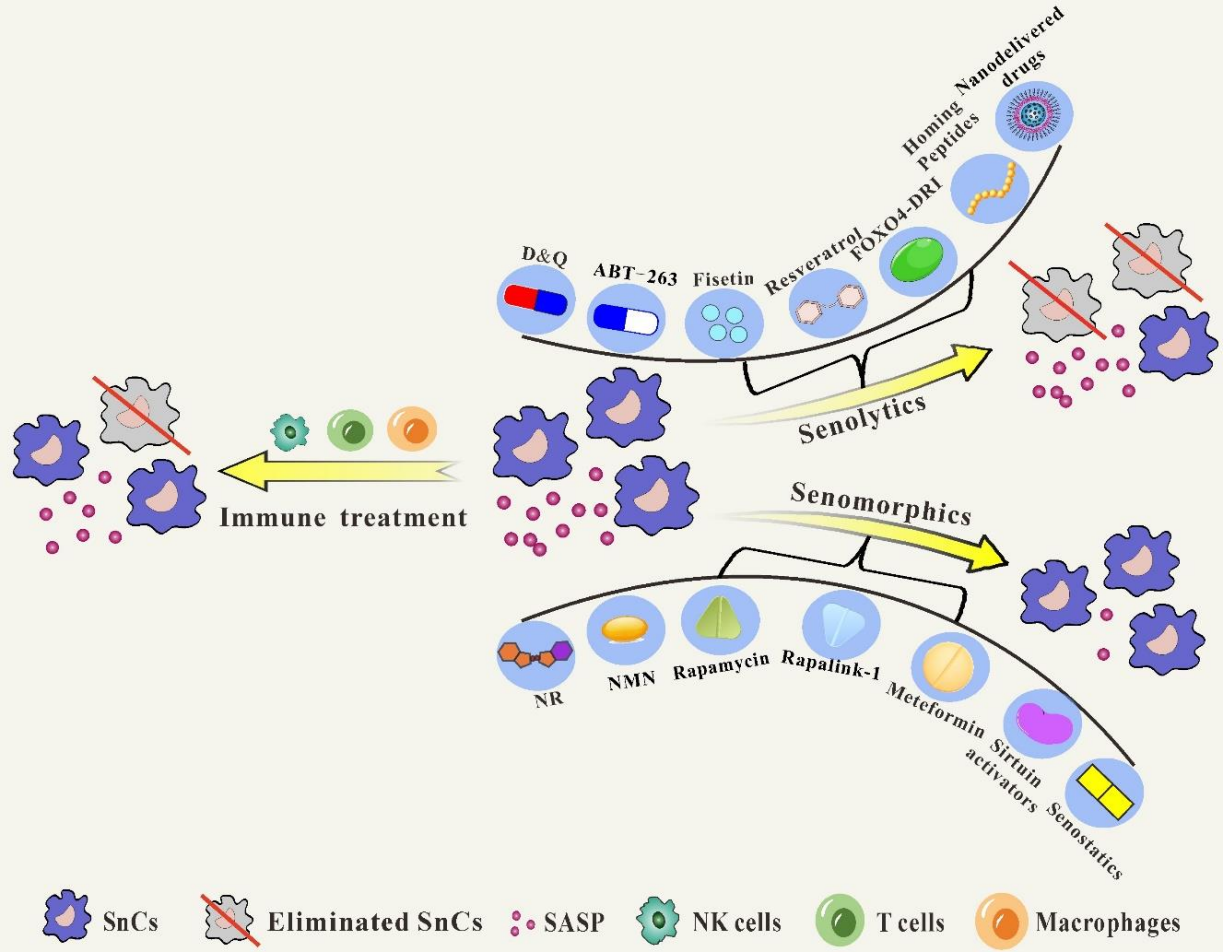
**Several major therapeutic strategies for targeting SnCs (e.g., senolytics, senomorphics, immune-based approaches)**. Senomorphics are compounds that modify the behavior of SnCs, while senolytics are compounds that selectively eliminate SnCs. The immune-based approaches allow specific recognition and removal of SnCs by active immune cells (mainly NK cells, T cells, and macrophages). Senolytics primarily includes D&Q, ABT-263, fisetin, resveratrol, FOXO4-DRI, homing peptides, nanodelivered drugs, etc. Senomorphics mainly have NAD^+^ boosters, mTOR inhibitors, metformin, sirtuin activators, senostatics, etc.

Immune cells can be modified to recognize and respond to disease states and act as "living drugs" when transferred to the patient. Methods of immune cell engineering, including pharmacological manipulation and genetic modification, have been reported for both in vitro and in vivo therapies. The use of engineered immune cell therapy has been reported clinically, and several engineered T cell therapies have been approved for the treatment of hematologic malignancies. So how do the immune system and engineered immune cells specifically recognize and remove SnCs? This is related to the monitoring of SnCs by different immune cells under different physiological and pathological conditions. SnCs are immunogenic through the expression of stimulatory ligands such as MICA/B that bind to NKG2D and activate their killing by NK cells [277, 379]. In addition, by secreting chemokines and cytokines, SnCs can recruit immune cells to tissues capable of removing SnCs [380]. Increased recruitment of CXCL9 and cytotoxic CD4^+^ T cells (CD4-CTL) correlates significantly with a decrease in senescent fibroblasts in aged skin, and cytotoxic CD4-CTL eliminates SnCs by targeting cytomegalovirus antigens [380]. Senescent fibroblasts express human leukocyte antigen class II (HLA-II) and human cytomegalovirus glycoprotein B (HCMV-gB), which become direct targets of CD4-CTL [380]. Innate CD4-CTL of the skin eliminates HCMV-gB^+^ senescent fibroblasts in an HLA II-dependent manner, and HCMV-gB activates CD4 from human skin [381].

The role of immunotherapy in aging and age-related diseases is also a current concern. In one study, intracerebroventricular injection of Aβ-specific T cells in AD mice induced major histocompatibility complex (MHC) class II expression on microglia, and these MHC class II-positive microglia had a neuroprotective phenotype and enhanced plaque clearance [382], which highlights the role of mutual signaling between immune cells. In one study, higher plasma IL-12p70 and INF-γ levels in cognitively normal individuals were associated with reduced future cognitive decline [383]. IL-12p70 polarizes T cells into a proinflammatory INF-γ-producing T cell subpopulation, suggesting that T cells in this subpopulation may have a protective effect [382]. In a paranellular effect mediated by SASP, SnCs can remodel surrounding tissues by modulating the characteristics of neighboring cells (e.g., stromal cells, immune cells, and cancer cells), such as the senescence-monitoring effects (tumor suppression) and suppression of antitumor immunity observed in the majority of senescent cancer-associated fibroblasts and senescent T cells (tumor-promoting) [384]. How does SASP attract, recruit, and activate these immune cells? The senescence phenotype can be mediated through SASP-mediated paracrine or direct cell contact pathways [48], providing further complexity to the senescence microenvironment. "Local" inflammatory cytokine signaling from SnCs can be amplified in a proximal manner [385]. However, it is unclear how SnCs communicate with more distant circulating immune cells, which are normally separated from parenchymal cells through the vascular endothelium. Immune cells eliminate senescent liver cells, thereby preventing the development of malignant tumors. HCC is consistent with the hypothesis of cellular senescence as a protective mechanism for cancer, finding that the abolition of the senescence program due to additional cancer-causing mutations, such as p53 mutations, leads to the development of aggressive HCC [386]. However, the restoration of p53 in liver tumors can trigger immune cell-mediated clearance of senescent tumor cells [387]. There are also examples of treating age-related diseases through the use of immunotherapy in combination with other approaches. In an experimental model, NF-κB and mTOR coordinated radiation-induced CXCL8 secretion in cancer cells with senescence characteristics, leading to targeted migration of CD56-dim NK cells, thus linking senescence-associated CXCL8 release to innate immune surveillance of human tumors. This suggested that the combination of NK cells and radiotherapy is a plausible strategy for cancer treatment [388]. From the above, we see the prospect of combining immunotherapy with other methods in the treatment of cancer. At present, there are many immunotherapies for SnCs, but how to find a highly effective targeted drug is still an urgent problem to be solved (Fig. 2).

## 9. Challenges and Considerations

With the deepening of our understanding of the characteristics of SnCs in vivo and in vitro, we can see that there are still many challenges on the road to anti-aging targeting SnCs. Cellular senescence is a dynamic process, and it is still unclear how many "senescence phenotypes" exist among different cell types at the single-cell level [43]. At the same time, there may be a high degree of heterogeneity under the stimulation of different-induced aging, which makes the anti-aging path more complicated under the action of multiple comprehensive factors [43]. A major limitation in the anti-aging field is the lack of a single, universal, or model-specific biomarker to identify SnCs in culture or tissue samples, for which the identification of SnCs relies on the identification of multiple combinations of markers to differentiate between stably stagnant SnCs and their quiescent or differentiated counterparts [43]. Currently, single-cell transcriptomics approaches, including spatial transcriptomics, are the only option to fully understand the complexity of SnCs and identify similar cells. Therefore, finding better methods that can distinguish between subtypes of SnCs and identify the triggers of senescence for each subtype will allow us to identify specific subpopulations of SnCs that may be most deleterious to tissue function and can be more precisely targeted to the affected area, which will greatly optimize both senolytic and senmorphic approaches while minimizing deleterious effects [43]. In addition, reliable markers of aging can be measured noninvasively in blood or urine [389]. Recently, urinary levels of α-Klotho, an endocrine transmembrane protein thought to inhibit senescence, were shown to reflect the associated mouse brain and kidney SnCs load [390]. It has also been found that anti-aging drug treatment restored renal α-Klotho levels by testing urine from mice and patients with IPF [390].

In addition, there is the problem of being off-target. SnCs are heterogeneous in terms of transcription, metabolism, and SASP profiles on a species, cell type, and even individual cell basis, resulting in different dependencies on various aging pathways. To further complicate matters, not all metabolic changes associated with senescence are SnCs-specific. Terminally differentiated and post-mitotic cells, such as neurons, cardiomyocytes, adipocytes, and other cells, also exhibit senescence-associated phenotypes, such as increased expression of p16INK4a or other CDKIs, which may not reflect a true SnCs state. In addition, specific targeting of shared signaling pathways between SnCs and non-SnCs may lead to off-target effects that hinder clinical applications. For example, navitoclax, a Bcl-2 family inhibitor, not only has a senolytic effect on certain cell types but also exhibits severe cytotoxic effects on platelets and neutrophils, resulting in thrombocytopenia and neutropenia [43]. In addition, in some cases, phagocytes may fail to recognize senescent or apoptotic cells, rendering the senolytics approach deleterious as the lack of cytophagocytosis can also lead to secondary cellular necrosis, which can result in pro-inflammatory injury and failure of component breakdown [391]. Monitoring the status and function of SnCs can help scientists better understand the aging process, reveal the association between aging and age-related diseases, and provide important clues for the development of intervention strategies against aging. Recently, newly developed imaging-based tools and fluorescent tracers to monitor real-time senescence load and the therapeutic activity of senescence therapies in the clinic have gained extensive attention from researchers [392-394], which may provide more possibilities for anti-aging therapies targeting SnCs.

To design anti-aging treatments for specific diseases, we need to better understand which cell types senesce and, more importantly, why eliminating certain SnC types can be detrimental [348]. The use of senolytics as anti-aging agents to selectively clear SnCs is a novel therapeutic model. However, translating anti-aging drugs into clinical interventions for human application will face many difficulties and challenges. The first and foremost problem is the ethical issue of clearly understanding the long-term and short-term side effects faced by removing SnCs [68]. Previously, we needed to conduct clinical tests to observe whether patients with serious diseases (such as IPF, diabetic complications, diastolic heart failure, advanced osteoporosis, dementia, or infection with COVID-19) had any significant side effects after being treated with anti-aging drugs [68]. It can be demonstrated whether the anti-aging drugs can reach the target site to kill the SnCs after being applied to the human body [68]. On the other hand, there is the issue of safety. Although anti-aging drugs have obvious benefits such as prolonging a healthy life and delaying, preventing, or treating various chronic diseases, it is still uncertain whether the follow-up problems caused by drug use are easy to solve, and more information about safety, tolerability, side effects, and effectiveness in reducing SnCs is needed [395]. When targeting SnCs, safety issues may become a major challenge due to the physiological role of SnCs and the side effects of existing senolytics, and some anti-aging agents, especially Bcl-2 family inhibitors, pose a cytotoxic threat when administered to non-SnCs [396]. Therefore, we believe that the use of anti-aging agents should be applied in the clinic after careful monitoring and strictly controlled clinical trials.

Each individual has different physical qualities, health conditions, and differences in how the drug works. Taking the mTOR inhibitor rapamycin as an example, several studies have shown that the efficacy of rapamycin is also different for different genders, and its life-extending effect is more pronounced in women than in men [234]. Therefore, this also reveals the importance of personalized and precise delivery of anti-aging drug therapy. Aging can be targeted from multiple angles (promoting, preventing, killing, regulating, and clearing). The key is that we need to look at the essence of the phenomenon, determine what drugs we should use, when to give them, and how to target aging. Whether it is beneficial or harmful, we need to make decisions after finding out its mechanism [391]. Many advances have been made in recent biomedical research, and many mechanisms have been explained on the basis of pathology, but there are still some pathologic mechanisms that remain unaccounted for, and a single approach is still not a good solution to all problems. In fact, many neurodegenerative diseases, such as AD and PD, are clinically heterogeneous diseases with a strong genetic component, and even though they share the same pathological features, their clinical course is different, which also determines their therapeutic approaches to be different [397]. Therefore, the clinical evaluation and monitoring of SnCs and secreted related biomolecules is of great significance for the prevention, diagnosis, and treatment of aging and age-related diseases, which can provide physicians with more accurate information and individualized treatment plans to improve the health status and quality of life of patients. Intervention studies for aging are evolving and include a variety of approaches such as drug therapy [398], gene therapy [399], and cell therapy [400]. By monitoring changes in SnCs, the efficacy and safety of these intervention strategies can be evaluated, providing a basis for the optimization of clinical treatment. The aging process varies among individuals, and each person ages at different rates and in different ways [401]. By monitoring the characteristics of SnCs, guidance can be provided for individualized treatment, and corresponding prevention and treatment strategies can be formulated according to the aging characteristics of individuals. Early detection and intervention of abnormal changes in SnCs can provide early warning signals and contribute to the early diagnosis and prevention of aging and age-related diseases.

## 10. Future Directions and Research Gaps

In order to target SnCs for effective therapy, these cells first need to be accurately identified and labeled. Researchers are exploring a variety of methods and labeling techniques to be able to reliably identify and localize SnCs. Given the wide availability of second-generation sequencing, there has been considerable interest in monitoring the response to senolytic therapy [401]. However, this has been challenging, especially at the single-cell level [402]. This is partly due to inaccurate definitions of heterogeneous populations of SnCs and their associated SASP, which complicates monitoring of SnC clearance [403]. Finding a reference standard for the rate of biological senescence is key to understanding the molecular mechanisms involved in the aging process, and defining and validating this metric in humans would open a new door for the field of anti-aging medicine, which would overcome the limitations of current disease definitions and take a global view of health with a greater focus on preventive measures in the face of aging [404].

Currently, assays for SnCs include the measurement of multiple senescence markers in tissue lysates. However, there is no single characteristic marker for SnCs, so it is necessary to analyze them from a single-cell perspective to fully characterize the heterogeneity of SnCs. Therefore, there is a need to find new tools and methods to detect SnCs in vivo and isolate them in order to advance clinical trials. We need in-depth phenotyping of SnCs by genomics, proteomics, lipidomics, and metabolomics to better define the secretory phenotypes, dynamic properties, and physiological effects (good and bad) of aging and SnCs [27].

With rapid advances in the biology of aging, the identification and assessment of interventions for human longevity has become a key goal in the field. Biomarkers of SnCs in realistic time frames are crucial tools to achieve these goals. However, there is a lack of standards and consensus on the characterization of reliable aging biomarkers, which hinders their further development and validation for clinical applications [405]. In the context of an aging global population, a comprehensive understanding of aging is a critical first step in treating age-related diseases and delaying aging dysfunction. Future aging assessment studies will focus on combining disease characteristics with aging markers, utilizing more practical detection techniques for timely diagnosis at the early stage of disease, providing the possibility of early detection and prevention of age-related diseases, and providing a better theoretical basis and practical application value for improving the quality of human survival.

With SnCs-specific recognition identified, the next step is to develop strategies for clearing SnCs. Currently, common approaches include drugs and gene therapy, as well as the use of immunotherapy to direct the immune system to attack SnCs. However, further research and refinement of these techniques are needed to improve their effectiveness and safety. The potential for anti-aging drugs to extend lifespan while delaying, preventing, or treating a wide range of chronic diseases, geriatric syndromes, and age-related declines in physical resilience is uncertain, and more information is needed on safety, tolerability, side effects, and target engagement (effectiveness in reducing the burden of SnCs). Therefore, we believe that the use of anti-aging drugs should be closely monitored, with the prerequisite of placebo clinical trials as controls. More appropriate methods need to be found to optimize and identify new anti-aging therapeutics, improve the intermittent administration of senolytics, and improve their drug safety [27]. Although there is substantial preclinical evidence that senolytics therapy can treat multiple disease processes, it is unclear how senolytics therapy strategies can be applied to different SnC populations and translated into clinical outcomes, and whether there is a difference between the efficacy of senolytics being administered prophylactically or at the time of onset. Ideally, as our understanding of the heterogeneity of aging improves, it will be more likely in the future to use rationally designed strategies to target and eliminate the most deleterious subpopulations of SnCs (such as utilizing single-cell differential gene expression profiles of SnCs). However, these translational problems remain challenging given the inherent differences between animal models and humans themselves, which are characterized by complex immune functions and differences in the kinetics of SnC accumulation. Together, these gaps need to be filled by the burgeoning field of cellular senescence and are likely to be the focus of research for the foreseeable future [406]. From the establishment of a "death sequence" screening system to the discovery of clear targets and attenuated side effects of the ABT-199/birinapant combination for the removal of SnCs to the verification of these effects in mouse models [407]. The establishment of the "death sequence" system allows senolytics to move beyond "old drugs" such as cancer drugs, plant extracts, and metformin to a broader field of research and development [407]. It can be seen that the development of senolytics has been further advanced by clearer targets and fewer side effects [407].

Although targeted SnCs therapy has shown potential benefits in laboratory and animal models, evaluations of long-term effects and safety are still necessary. More clinical trials and long-term follow-up studies are needed to assess the long-term effects of these treatments as well as potential side effects and risks. Like many therapies, anti-aging therapies have their own conditions and limitations. We need to build better in vitro and in vivo models, find the aging-related diseases that are most likely to benefit from them, develop potential biomarkers that are relevant to the treatment of the diseases, screen appropriate patient populations, and ensure that these therapies are sufficiently safe and well-targeted. If we can address these issues, the future of the anti-aging field will be immense. In this age of aging, aging imposes a great burden on society, and anti-aging therapies are expected to allow people to live longer and stay healthy. Preliminary results suggest that senotherapy could be applied to humans. Because of their potential side effects, senolytic drugs must be used with caution. Although anti-aging drugs and their similar compounds have some potential to regulate aging and age-related diseases, we should be cautious about recommending them in clinical practice without sufficient clinical supporting studies [346].

Aging research has potential implications for personalized medicine and disease prevention. With a better understanding of the mechanisms of aging, we can better understand the relationship between aging and disease and develop more targeted treatment and prevention strategies. Individualized medicine is an emerging medical practice that utilizes an individual's genetic characteristics to guide diagnostic and therapeutic decisions related to disease prevention, diagnosis, and treatment [408]. A person's aging pattern depends on which physiological system in his or her body is aging the fastest. In the future, doctors can use this detected information to provide better advice and personalized medical treatment for patients' lifestyles [409]. With SnC identification and removal technologies, we can target individual SnCs to slow down the aging process and the development of related diseases [410]. In addition, aging is a risk factor for many diseases, including cardiovascular disease [411], cancer [412], diabetes [413], etc. Targeting SnCs may help reduce the risk of developing these age-related diseases, and by understanding the relationship between aging and these diseases, we can develop more effective prevention strategies [414]. In conclusion, targeting SnCs provides a new direction for anti-aging therapy, offering hope for prolonging healthy lifespans and improving the quality of life for older adults. With further development of technology and research, we expect to see more breakthroughs and applications of targeted SnC therapy. Aging research has potential implications for personalized medicine and disease prevention, supporting the development of more precise treatment and prevention strategies. With further research into the mechanisms of aging, we are expected to make greater breakthroughs in individual health management and disease prevention.

## 11. Conclusion

This paper provides insights into the importance of targeting SnCs in addressing aging and age-related diseases. Firstly, through a systematic review, we elucidate the key role of SnCs in a variety of diseases, such as neurodegenerative diseases, cardiovascular diseases, cancers, and metabolic diseases. At the same time, we highlight the current research progress in the field of targeting SnCs, including senolytics (selective elimination of SnCs) and senomorphics (prevention of harmful external effects of SnCs), which are two main types of compounds in current anti-aging therapies. The therapeutic mechanism and the latest research on these two kinds of compounds for aging and age-related diseases were introduced. In addition, we describe the therapeutic role of immune-based therapies targeting SnCs in aging and age-related diseases and point out therapeutic strategies targeting specific cell types and molecular markers, as well as the remarkable results of these strategies in animal experiments and clinical trials. Finally, this review further explores the potential clinical significance of the field of targeting SnCs, emphasizing the potential efficacy of targeting SnCs and the importance of this strategy for improving the quality of life and slowing the progression of age-related diseases in the elderly. We also reveal the possible challenges and improvement directions of therapies targeting SnCs in future clinical practice, and we firmly believe that targeting SnCs in the future will bring greater well-being to the elderly population. Overall, this review provides a comprehensive and in-depth overview of the significance of targeting SnCs in addressing aging and age-related diseases and provides a clear picture of the development and potential clinical implications of the field, providing a strong guide for future research and clinical practice.
